# Molecular Design Principles for Achieving High-Efficiency Light-Induced Charge Separation at the Nanometer Scale

**DOI:** 10.1021/jacsau.5c01092

**Published:** 2025-10-10

**Authors:** Mathis Brändlin, Felix A. Himmelreich, Oliver S. Wenger

**Affiliations:** Department of Chemistry, 27209 University of Basel, St. Johanns-Ring 19, Basel 4056, Switzerland

**Keywords:** electron transfer, artificial photosynthesis, solar energy conversion, photochemistry, laser spectroscopy

## Abstract

The light-induced separation of charges, fundamental to natural photosynthesis, is key to converting solar energy into chemical energy in artificial systems. One challenge is that charges tend to spontaneously recombine in a downhill, energy-wasting process. This recombination can be slowed by increasing the distance between charges, but doing so also reduces the efficiency of the initial charge-separating step. We investigated how the quantum efficiency of energy-storing charge separation and the rate of energy-wasting charge recombination vary over distances from 22 to 44 Å in three structurally well-defined molecular donor–photosensitizer–acceptor compounds. Our key finding is that separation efficiency can remain high, around 60%, even at distances of up to 44 Å when redox relays are used instead of passive molecular bridges. At the same time, undesirable charge recombination remains slow, occurring on a time scale approaching the low-millisecond range. These results support the widely accepted view that multistep hopping is more effective than single-step electron tunneling. However, while previous studies have strongly focused on kinetics and charge-transfer rates, the question of how the efficiency of light-induced charge separation in donor–photosensitizer–acceptor systems is affected by the transition from tunneling to hopping remains underexplored. The newly gained insight from focusing on quantum efficiencies rather than charge-separation rates is critically important, as light-independent charge recombination events often go undetected when only rates of light-induced steps are in focus. Overall, our findings provide valuable design principles for creating artificial photosynthetic systems with high light-to-chemical energy conversion efficiency, offering important insights for the broader fields of solar energy conversion and artificial photosynthesis.

## Graphical abstract

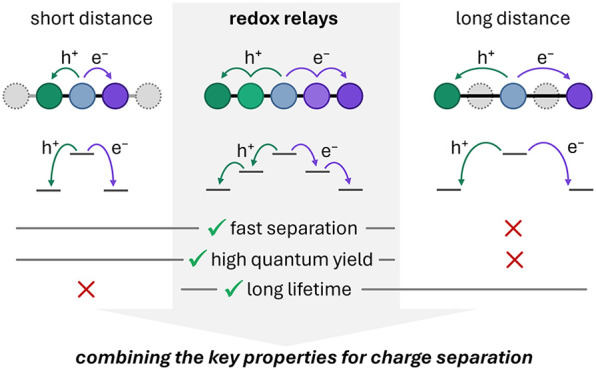

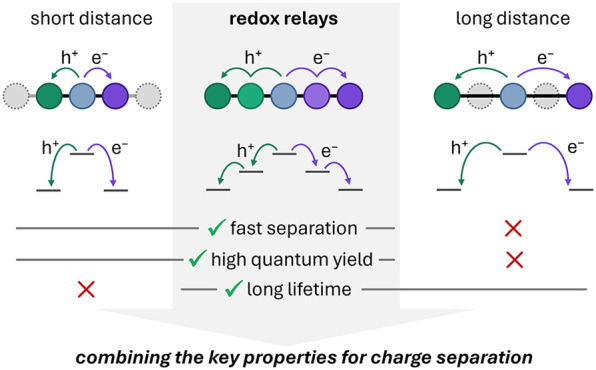

## Introduction

The initial steps of natural photosynthesis following light absorption involve a series of photoinduced electron transfer processes that lead to the separation of negative charges (electrons) from positive charges (commonly referred to as “holes” or electron vacancies). These separated charges are then transferred through a sophisticated redox chain.^1^ Multiple electrons are funneled toward the NADP^+^ reduction catalyst where they generate high-energy NADPH, an essential molecule that ultimately powers the reduction of CO_2_ into organic matter. Simultaneously, multiple holes are transferred and accumulated at the water oxidation complex, where they drive the splitting of water molecules. The charge separation network has evolved to include small energy gaps between individual redox relay stations, minimizing energy losses while working against the intrinsic tendency of the charges to recombine.

These processes are desirable to mimic in artificial photosynthesis to produce high-energy solar fuels with abundant sunlight.^2–4^ In an ideal system, once charge separation has occurred, the redox equivalents are transferred to and accumulated on suitable catalysts.^5–7^ Reducing equivalents are directed to a catalyst capable of converting a substrate such as protons or carbon dioxide into so-called solar fuels.^8–11^ In parallel, the oxidizing equivalents are used to drive water oxidation, thereby completing the redox cycle.^4^

Analogous to the absorption of a photon by chlorophyll in natural photosynthesis, the excitation of an artificial photosensitizer such as Ru­(bpy)_3_^2+^ (bpy = 2,2′-bipyridine) initiates charge separation. The excited state of the photosensitizer can be quenched by suitable electron donors or acceptors in bimolecular reactions, leading to the formation of photoredox products. These primary photoproducts can subsequently migrate apart by diffusion, ideally leading to terminal photoproducts that are stable on long time scales.^12^ This mechanism underlies many typical photoredox reactions.^13,14^

To study electron transfer processes in molecules on a more basic level and with artificial photosynthetic reaction systems in mind, it is beneficial to have more precise control over the charge-separating system. For this reason, electron donors and acceptors are often covalently linked, creating well-defined molecular systems^15,16^ that are also interesting for quantum information science.^17–19^ Recently, donor–photosensitizer–acceptor (D–PS–A) triads based on free porphyrins^20,21^ or porphyrins coordinating to Pd^II^,^22^ Zn^II^,^23,24^ and Al^III^ ^25,26^ were investigated, following many early works based on porphyrin photosensitizers.^27^

While such D–PS–A systems can provide fundamental insights about the influence of the electronic structure,^28^ driving force and solvent effects,^29–31^ and external magnetic fields^32,33^ or allow the vibronic manipulation of the bridge,^34–36^ they also keep the opposite charges in proximity to one another. This increases the likelihood of unwanted recombination. As in natural photosynthesis, the formation of solar fuels typically involves multielectron redox events, not just single-electron transfer processes.^37,38^ Therefore, charge accumulation is essential for driving useful fuel-forming reactions.^5,6,39^ This requires sufficient time for a second photon to be absorbed and for a secondary charge separation process to occur before the initially formed charge-separated state collapses.^40–42^ However, in most artificial D–PS–A systems known to date, the irradiance of sunlight is too low for a second absorption event to occur within the nanosecond to microsecond lifetime regime of typical primary charge-separated states.^6,39^

One strategy to extend these lifetimes is to increase the distance between the electron donor and acceptor.^43,44^ While the relationship between recombination and distance is more complex than just decreasing with increasing distance,^45^ it has been shown that increasing the spatial separation of electrons and holes can slow down charge recombination into the hundreds of microseconds to milliseconds regime.^46–48^ Solar irradiance allows for the absorption of a second photon on a time scale ranging from milliseconds to seconds, thereby enabling, in principle, the accumulation of both negative and positive charges.

Unfortunately, increasing the spatial separation between donors and acceptors comes with a significant drawback: It also slows down the initial charge separation process.^43^ As a result, the excited state has more time to decay before a charge-separated state (CSS) can form, leading to a substantially lower quantum yield for CSS formation. One way to maintaining a high quantum yield is to use large driving forces for charge separation, which can make electron transfer kinetically competitive with the decay of the photosensitizer’s excited state. However, this strategy reduces the amount of energy that can be stored in the CSS.^49^

An alternative strategy involves introducing redox relay stations similar to those found in natural photosynthetic reaction centers,^50,51^ which contain multiple redox-active components between the charge-accumulating sites. This allows individual electron transfer steps to proceed rapidly over relatively short distances (<15 Å), enabling a multistep hopping mechanism that is significantly more efficient than single-step tunneling.^44,52–55^ A number of previously reported systems, specifically four- and five-component assemblies known as tetrads and pentads, have been based on this design principle.^42,56,57^

However, two key questions remained unanswered:

How does the quantum yield for CSS formation (Φ_CSS_) depend on the donor–acceptor distance?
And to what extent is multistep hopping via redox relays superior to single-step tunneling for achieving efficient charge separation?

These are the critical gaps in knowledge that the research presented here seeks to address.

We recently reported the molecular D_2_–D_1_–PS–A_1_–A_2_ pentad in Figure 1c, in which we achieved vectorial separation of two electrons from two holes for the first time in a purely molecular system.^58^ While this is an important proof of concept and was the focus of our previous study, the underlying elementary processes involved in the primary charge separation, as well as the two key knowledge gaps mentioned above, remained largely unaddressed.

**Figure 1:**
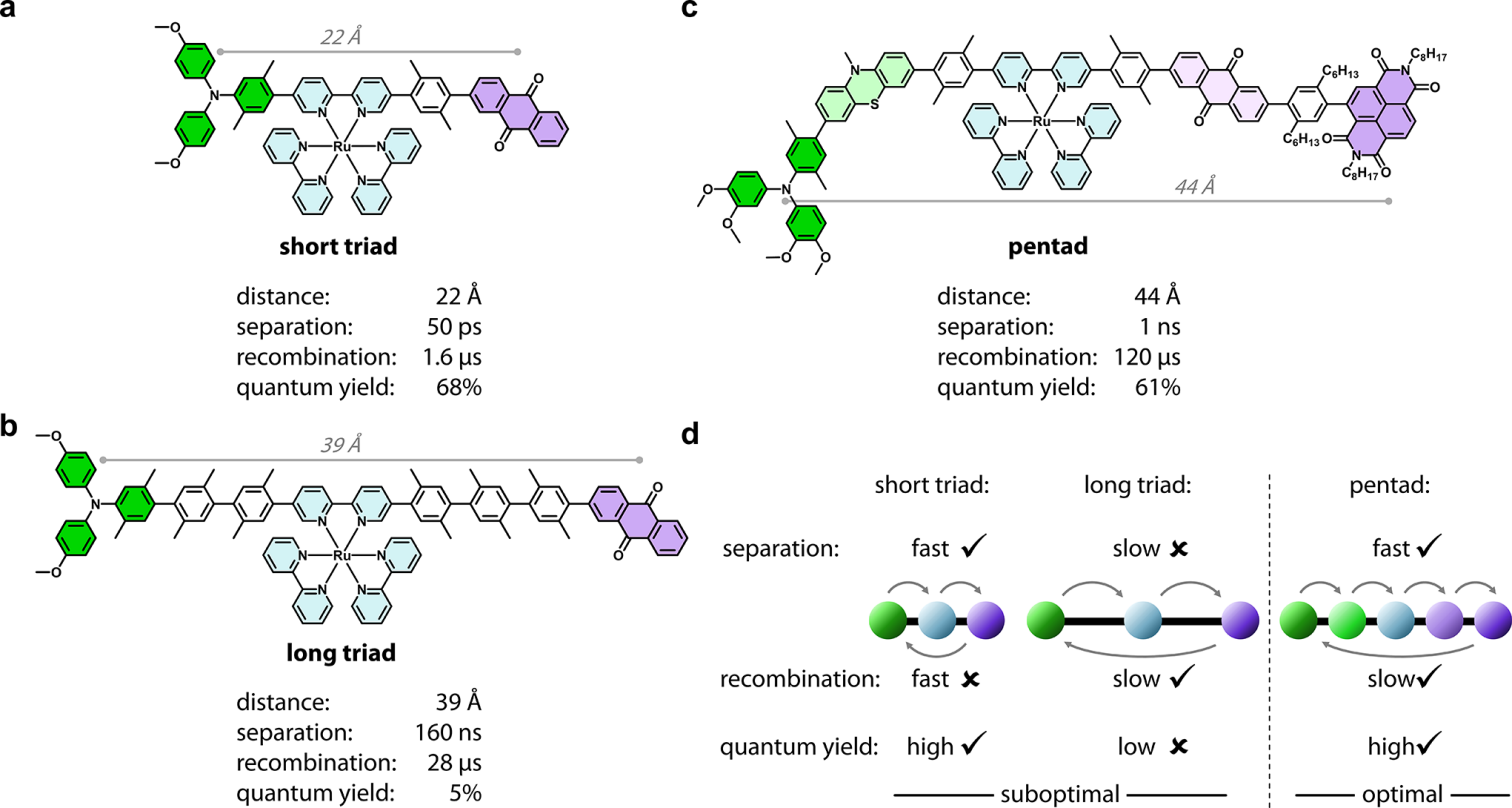
Structures of the three donor–photosensitizer–acceptor (D–PS–A) compounds studied here: (a) Short D–PS–A triad. (b) Long D–PS–A triad with multiple xylene units as spacers to increase the distance between the individual redox-active units. (c) D_2_–D_1_–PS–A_1_–A_2_ pentad that uses redox-active relay stations as spacers. The distances are obtained from force-field optimized models of completely stretched structures. The long molecules might possess some flexibility in solution resulting in variation of these distances. (d) Comparison of the three key performance parameters (charge separation kinetics, charge-separated state lifetime, quantum yield for formation of the charge-separated state) in the molecules in (a–c).

In this study, we compare the kinetics and quantum yield of the charge separation processes in our D_2_–D_1_–PS–A_1_–A_2_ pentad with those in two prototypical D–PS–A triads. In the short triad (Figure 1a), the donor and the acceptor are separated only by 22 Å (center-to-center), enabling rapid (50 ps) and efficient (Φ_CSS_ = 68%) charge separation, followed by spontaneous charge recombination with a 1.6 μs time constant. When the donor–acceptor distance is extended to 39 Å in the long triad (Figure 1b) by inserting additional redox-inactive xylene spacers, all performance metrics change significantly. Charge separation slows by three orders of magnitude (160 ns), and the quantum yield Φ_CSS_ drops sharply to 5%. Although charge recombination is also slowed, occurring with a 28 μs time constant, this benefit is outweighed by the poor efficiency of the initial charge separation step. In contrast, the molecular pentad with a donor–acceptor distance of approximately 44 Å (Figure 1c), about 5 Å longer than in the long triad, achieves a high quantum yield (61%) compared to the short triad,^59^ while also producing a longer-lived charge-separated state (120 μs) than either triad. This striking result highlights the role of the redox-active spacers D_1_ and A_1_, which enable efficient multistep hopping during charge separation but do not facilitate charge recombination. This distinction, and its impact on performance (in particular quantum yields), has to our knowledge not been demonstrated with such clarity in previous studies of artificial D–PS–A compounds.

## Results and Discussion

### Molecular Design and Synthesis of the Pentad

Early studies of D–PS–A molecules focused heavily on porphyrin-based photosensitizers.^27,60,61^ However, we and others have found it advantageous to use Ru^II^ polypyridines or cyclometalated Ir^III^ complexes as photosensitizers. These transition metal complexes are relatively easy to incorporate into extended covalent molecular architectures,^62–67^ exhibit favorable excited-state properties, and possess distinct spectroscopic signatures in both their excited states as well as in their oxidized and reduced forms. These features make them particularly well suited for monitoring charge-transfer processes by using transient UV–vis absorption spectroscopy.

Anthraquinone (AQ)^30,43,67–69^ and naphthalene diimide (NDI)^32,33,42,47,70–72^ are commonly used for temporary electron storage in D–PS–A molecules. Here, they were chosen as electron acceptors because they differ sufficiently in their reduction potentials, which is an important property to build a well-defined redox gradient without relevant back electron transfers. Although NDI is often *N*-connected, it was introduced here via a core connection. This has been shown to facilitate faster electron transfer.^71,72^

As electron donors, tertiary amines provide tunable properties that can be easily adjusted through chemical modifications. For example, often used phenothiazines^73–77^ can be modulated through *N*-functionalization^78,79^ and can be easily incorporated at both ends into a redox chain. Also, triarylamines (TAAs) are widely used as reversible electron donors and are frequently introduced into covalently linked triads.^41,43,68,80^ Beneficial features are their ease of installation and the distinct spectroscopic signatures of the radical cations formed upon oxidation. Their oxidation occurs at relatively low potentials and can be finely adjusted through substituents on the phenyl rings. A sufficient redox gradient was achieved by combining a moderately electron-rich methylphenothiazine (PTZ) with electron-rich veratroles on TAA, thereby making TAA a stronger electron donor than PTZ.

The 1,4-dimethylphenyl (xylyl) bridges sterically induce twisting, which decouples the individual redox-active units electronically.^81–83^ Directly next to NDI, the methyl groups are replaced by *n*-hexyl chains to prevent stacking of multiple NDI units, which could lead to solubility problems.^84^

To build the chain of linked functional units on a single 2,2′-bipyridine (bpy) ligand for Ru^II^, the individual building blocks were first synthesized and then connected with Suzuki–Miyaura cross-coupling reactions (Scheme 1).^58^ The synthesis of monobrominated NDI (**6**) involved unselective bromination of naphthalene dianhydride followed by diimide formation. At this stage, unbrominated NDI and dibrominated NDI-Br_2_ can be separated by careful normal-phase flash column chromatography.^72^

**Scheme 1:**
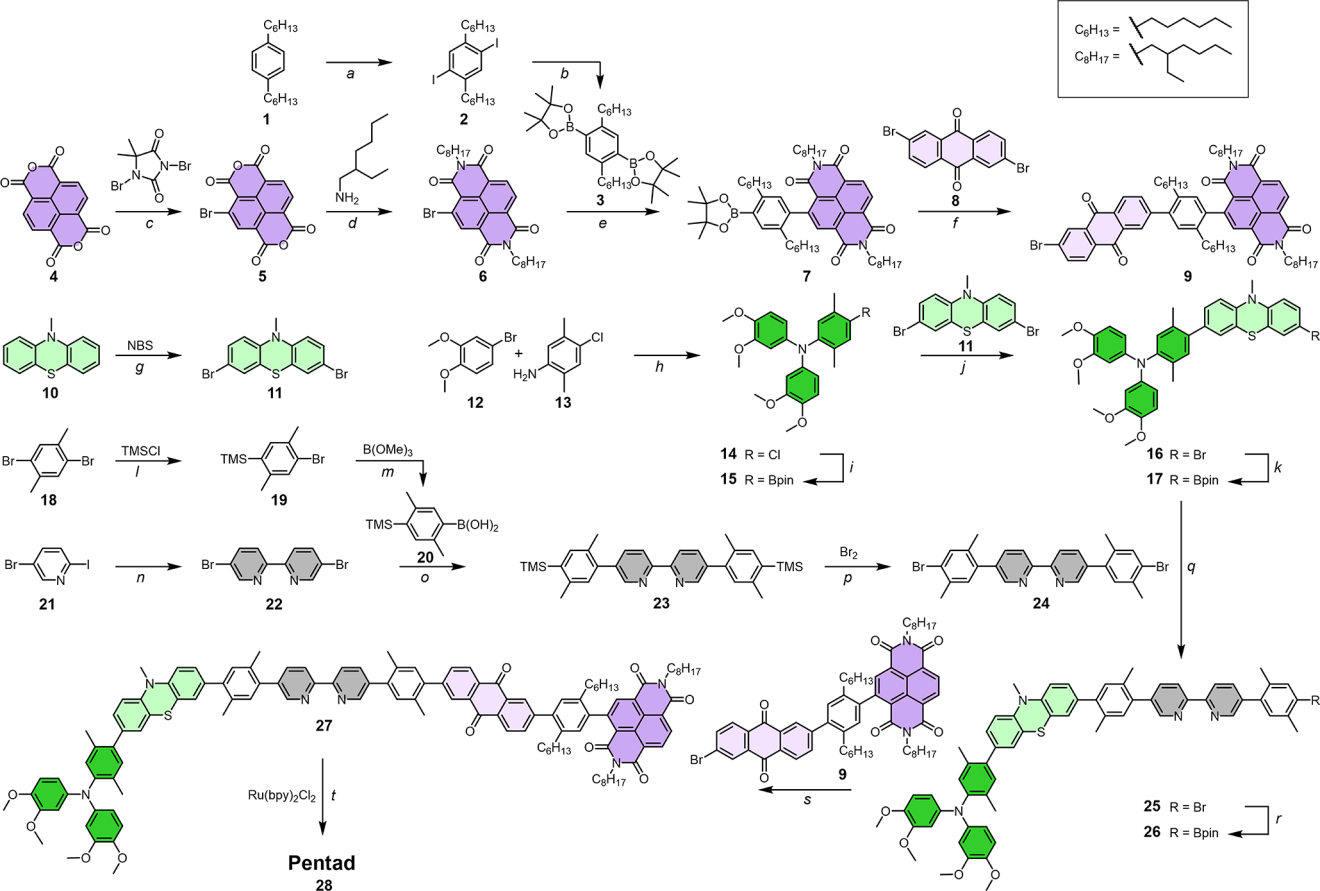
Synthesis Scheme of the Pentad^a^

a Reaction conditions: (a) I_2_, KIO_3_, H_2_SO_4_, AcOH, H_2_O. (b) B_2_pin_2_, Pd­(dppf)­Cl_2_, KOAc, 1,4-dioxane. (c) H_2_SO_4_. (d) AcOH. (e) Pd­(PPh_3_)_2_Cl_2_, Na_2_CO_3_, THF/H_2_O. (f) Pd­(PPh_3_)_2_Cl_2_, Na_2_CO_3_, THF/H_2_O. (g) CH_2_Cl_2_. (h) Pd_2_(dba)_3_, P­(*t*-Bu)_3_, *t*-BuOK, toluene. (i) B_2_pin_2_, Pd_2_(dba)_3_, PCy_3_, KOAc, 1,4-dioxane. (j) Pd­(PPh_3_)_4_, K_3_PO_4_, THF/H_2_O. (k) B_2_pin_2_, Pd­(dppf)­Cl_2_, KOAc, 1,4-dioxane. (l) *n*-BuLi, THF. (m) *n*-BuLi, THF. (n) (*n*-Bu)_6_Sn_2_, Pd­(PPh_3_)_4_, toluene. (o) Pd­(PPh_3_)_4_, Na_2_CO_3_, THF/H_2_O. (p) KOAc, CH_2_Cl_2_ (q) Pd_2_(dba)_3_, XPhos, Na_2_CO_3_, THF/H_2_O. (r) B_2_pin_2_, Pd­(PPh_3_)_4_, KOAc, DMF. (s) Pd­(PPh_3_)_4_, K_3_PO_4_, THF/H_2_O. (t) EtOH/CHCl_3_.

The directional chain nature of the rigid rod-like donor–acceptor construct incorporating a bpy ligand in its middle requires introducing asymmetry at multiple connections. Protection–deprotection strategies, for example those used for the synthesis of the central bipyridine unit **24**, were omitted whenever possible to decrease the number of reaction steps. Instead, monofunctionalization was achieved preferably by using one reactant in large excess followed by separation of the monofunctionalized product from excess starting material and the doubly coupled side product. However, for the synthesis of **7**, using an eightfold excess of the diborylated spacer **3** was not enough to sufficiently prevent twofold coupling of the reactive bromide **6**. Using a less active palladium catalyst (Pd­(PPh_3_)_2_Cl_2_) and conducting the reaction at unusually low temperatures (0 °C to room temperature) finally provided the desired product in 79% yield.

Using a less active catalyst at lower temperatures was not satisfactory for the subsequent reaction of **7** with 2,6-dibromoanthraquinone (**8**). We think that the poor solubility of **8** in the standard THF/H_2_O mixture at lower temperatures (40 °C) prevented a sufficient effective excess of the dibromide in solution. Moreover, the coupling reaction with **7** increases the solubility of the anthraquinone, making the second cross-coupling event more likely. To ensure an effective excess in solution, the reaction mixture was preheated to 40 °C before the addition of Pd­(PPh_3_)_2_Cl_2_, and the boronic ester **7** was added in three portions at 2 h intervals. However, the reaction yield could only be improved to ca. 40%, whereby doubly coupled NDI-hxy-AQ-hxy-NDI (hxy = 1,4-dihexylbenzene) was the only side product observed in relevant quantities.

The borylation of the electron-rich triarylamine **14** provides a large fraction of presumably protodeboronation product TAA-H, as described in literature.^43^ At this stage, chromatographic separation of this byproduct was unsuccessful. However, it was easily removed after the subsequent cross-coupling reaction and used as a reference compound. All the following cross-coupling reactions proceeded smoothly, without complications or unexpected behavior. The final pentad was isolated and purified on a 16 mg scale.

From the synthesis route in Scheme 1, it becomes obvious that the implementation of relay units comes at the cost of additional reaction steps. It is a compromise between synthesis efficiency and photophysical performance. The convergent approach used here, however, allows to functionalize the individual units before incorporation into the final ligand, decreasing the overall additional effort to some extent.

### Establishing the Redox Cascade

The electrochemical properties of the pentad, its primary ligand, and suitable reference molecules (see Figure S1) were characterized using cyclic voltammetry (Figure 2b and Figure S3). To ensure solubility and comparability, all measurements were conducted in Ar-saturated CH_2_Cl_2_ and the resulting reduction potentials are summarized in Table 1. These values are consistent with literature reports and confirm the intended redox gradient in the molecular pentad.

**Table 1:** Redox Potentials of the Individual Units in the Reference Compounds, the Main Ligand and the Pentad in Argon-Saturated CH_2_Cl_2_ in V *vs* SCE

redox couple	reference	ligand	pentad
TAA^•+/0^	0.61 V	0.60 V	0.60 V^a^
PTZ^•+/0^	0.73 V	0.78 V	0.73 V^a^
AQ^0/•–^	–0.99 V	–1.00 V	–0.92 V
NDI^0/•–^	–0.69 V	–0.72 V	–0.69 V
Ru(bpy)_3_^2+/+^	–1.33 V^a^ ^,^ ^85^		–1.35 V
*Ru(bpy)_3_^2+/+^	0.77 V^a^ ^,^ ^85^		
Ru(bpy)_3_^3+/2+^	1.29 V^a^ ^,^ ^85^		
*Ru(bpy)_3_^3+/2+^	–0.81 V^a^ ^,^ ^85^		

a In MeCN.

**Figure 2:**
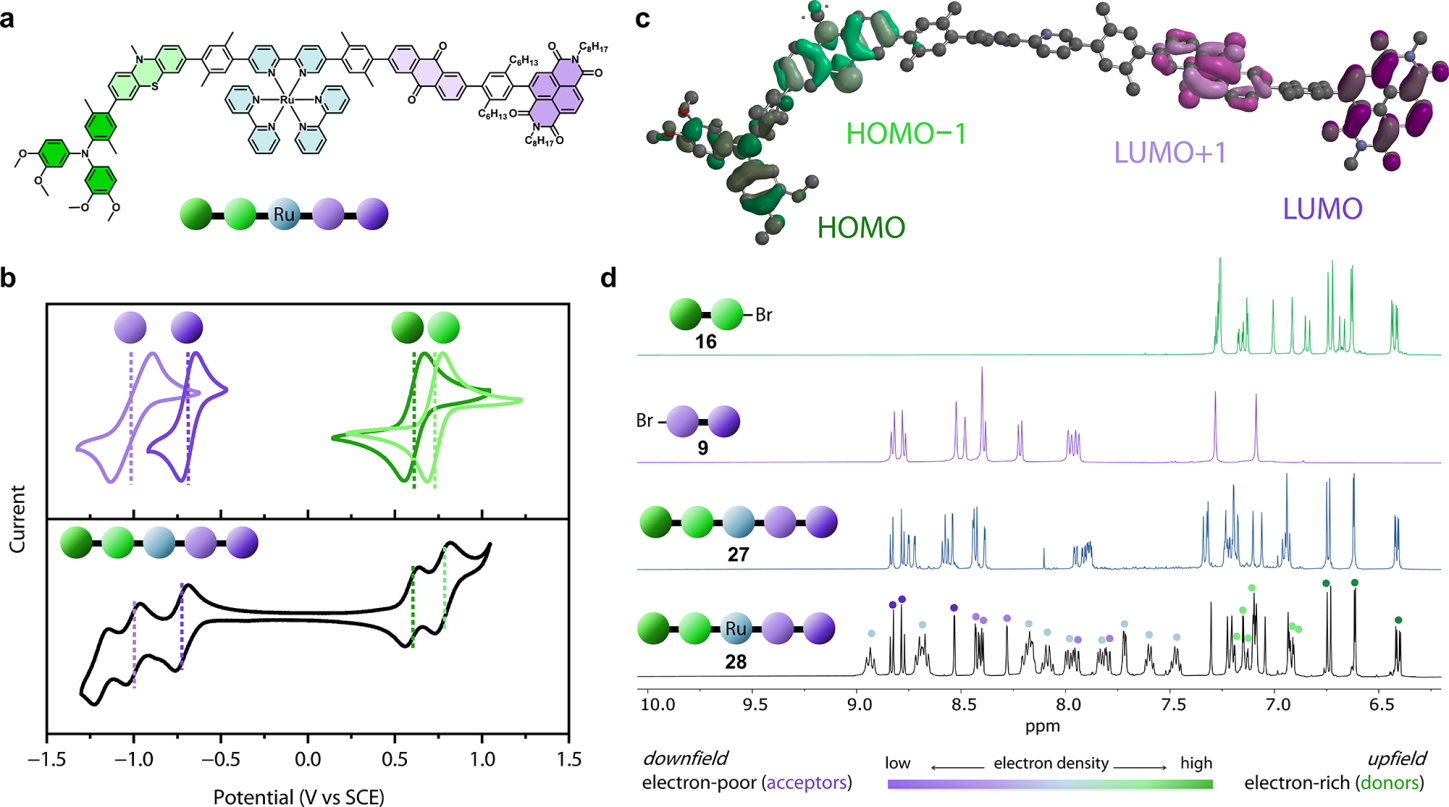
(a) Explanation of the simplified representation of the pentad used to illustrate the molecules and individual redox-active components investigated in (b–d). (b) Cyclic voltammograms of xylene-capped reference molecules for the individual redox units (top) and the main ligand of the pentad (bottom) in dry, Ar-saturated CH_2_Cl_2_. The molecular structures of the individual reference molecules can be found in Figure S1. (c) HOMO–1, HOMO, LUMO, and LUMO+1 obtained from DFT calculations (B3LYP, 6-31G*, alkyl chains omitted for clarity) on the molecular structure of the uncoordinated (free) ligand **27** incorporating the two donors and the two acceptors. The two highest occupied orbitals are expected to be oxidized first, while the two lowest unoccupied orbitals are the first to be reduced. Both the relative order of HOMO and HOMO–1 as well as the order of LUMO and LUMO+1 suggest the redox chain to work. The clearly localized molecular orbitals indicate sufficient electronic decoupling of the individual redox units. (d) ^1^H NMR spectra of the aromatic region of the donor branch (**16**), the acceptor branch (**9**), the ligand (**27**), and the pentad (**28**) either in CDCl_3_ (**16**) or CD_2_Cl_2_ (**9**, **27**, and **28**). In the bottom spectrum, the sets of signals are assigned to the respective redox units.

The long-lived ^3^MLCT (metal to ligand charge transfer) excited state of the sensitizer is well characterized and lies about 2.1 eV above the electronic ground state.^85^ This excited state is reduced at a redox potential of +0.77 V vs SCE in MeCN and oxidized at −0.81 V vs SCE.^85^ With the values from Table 1, reductive quenching of the ^3^MLCT excited state by PTZ has a very small driving force (Δ*G*_ET_ = −0.04 eV). However, this process can be coupled to a follow-up electron transfer from bpy^•–^ to AQ, which is exergonic by ca. −0.3 eV, generating an intermediate charge-separated state (CSS-1) that consists of D_2_–D_1_^•+^–PS–A_1_^•–^–A_2_ and stores an energy of ca. 1.7 eV (Figure 3b). Oxidative quenching of the excited state by AQ is endergonic by ca. 0.2 eV and, therefore, unlikely to occur. When comparing the redox potentials of D_2_ and D_1_, we can see that onward transfer of the hole is exergonic (Δ*G*_ET_ = −0.15 eV). Also on the acceptor branch, an exergonic onward charge transfer can be expected, transferring the electron from AQ^•–^ to NDI (Δ*G*_ET_ = −0.3 eV). After these two spontaneous secondary electron transfers, a charge-separated state CSS-2 consisting of D_2_^•+^–D_1_–PS–A_1_–A_2_^•–^ and storing ca. 1.3 eV is expected (Figure 3b). Because the secondary electron transfers require energy, the final charge-separated state stores less energy than the two triads from Figure 1, which store ca. 1.6 eV.

To visualize the localization of the molecular orbitals relevant to the redox chain, DFT calculations were performed on the ligand using the B3LYP/6-31G* level of theory (Figure 2c). The HOMO, expected to be located primarily on the terminal electron donor, is clearly localized on the TAA unit, while the HOMO–1, corresponding to the second strongest donor, is localized on the PTZ moiety. A minor contribution is observed on the adjacent xylene spacer. A similarly well-defined localization is found for the acceptor orbitals: The LUMO, expected to be located predominantly on the terminal electron acceptor, is localized on the NDI unit, whereas the LUMO+1 resides on the AQ moiety.

This redox gradient between the individual components of the pentad is also evident in ^1^H NMR spectroscopy. The proton signals of the donors resonate between 6.4 and 7.3 ppm (Figure 2d, green), while those of the acceptors resonate between 7.9 and 9.0 ppm (Figure 2d, violet). The singlet signals of the aromatic protons of the xylene bridges are found between ca. 6.9 and 7.3 ppm. The low chemical shifts of the donor branch arise from the high electron density of the electron-rich amines and the associated shielding of the hydrogen nuclei. On the other hand, the low electron density on the aromatic ring leads to low shielding and high shifts due to the electron-withdrawing nature of the carbonyls (AQ) or the imides (NDI) of the electron acceptors.

The chemical shifts of these signals remain largely unaltered when in the main ligand the donor branch and the acceptor branch are connected to the central bpy unit (Figure 2d, blue). Some of the signals of the inner D_1_ and A_1_ slightly shift due to the electron-withdrawing bipyridine.

In the ^1^H NMR spectrum of the pentad (Figure 2d, black), the additional two sets of bipyridine signals of the Ru­(bpy)_3_^2+^ unit can be seen (light blue dots). Apart from those, the chemical shifts of the redox units are largely unchanged compared to those of the free ligand. When assigning them to the individual redox units, they provide a clear gradient across from the most electron deficient NDI (dark violet dots) to the most electron-rich TAA (dark green dots).

### Watching the Charges Separate

The UV–vis absorption spectrum of the pentad in the electronic ground state (Figure S2) shows the expected MLCT absorption band at 450 nm. Below 400 nm, it features the maxima of the individual redox units. Only the Ru­(bpy)_3_^2+^ component of the pentad absorbs in the visible spectral range.

Photoinduced charge separation was investigated upon excitation into this MLCT band by transient absorption spectroscopy with picosecond time resolution. Excitation of the pentad with laser pulses of 250 fs length and a wavelength of 460 nm results in the fast formation of transient absorption signals corresponding to the ^3^MLCT excited state of Ru­(bpy)_3_^2+^ (Figure 3a). After 1 ps (first signal above the baseline), the rise of transient absorption signals with maxima at 440 and 600 nm and a shoulder at 670 nm can be observed. The rise of these features are interpreted as the quenching of the ^3^MLCT excited state and the associated formation of the initial charge-separated state CSS-1. After ca. 100 ps, these signals start to evolve into new bands with maxima at 390, 480, 600, 690, and 770 nm. This is in line with the stepwise model outlined in Figure 3b and the redox chain explained above. With this time resolution, it was not possible to disentangle whether D_1_^•+^ or A_1_^•–^ forms first. However, from the redox potentials given in Table 1, we expect reductive quenching of the excited state, as oxidative quenching by an electron transfer from photoexcited Ru­(bpy)_3_^2+^ to AQ is uphill by ca. +0.2 eV (Table 1). Hence, the initial electron transfer likely forms a TAA^•+^/Ru­(bpy)_3_^+^ pair, followed by electron transfer from Ru­(bpy)_3_^+^ to AQ (Δ*G*_ET_ = −0.3 eV). This coupled second electron transfer or a concerted mechanism^86^ could be the reason for the fast formation of CSS-1, despite the rather small driving force for excited state quenching. Finally, the electron shifts from AQ^•–^ to NDI (Δ*G*_ET_ = −0.3 eV), and the hole is transferred from PTZ^•+^ to TAA (Δ*G*_ET_ = −0.15 V).

**Figure 3:**
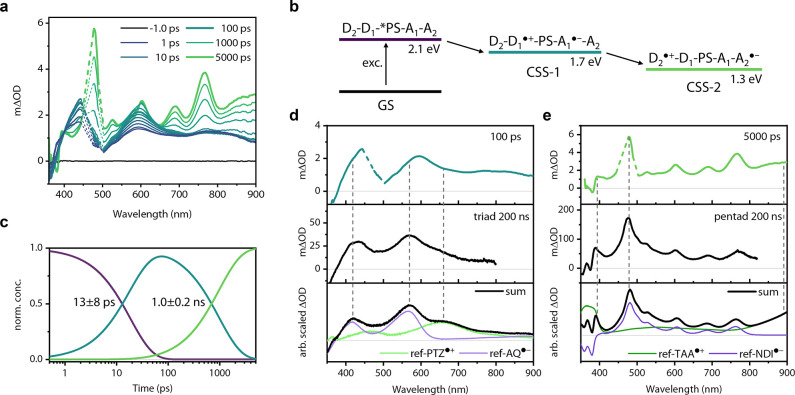
(a) UV–vis transient absorption spectra with varying delays of a 300 μM sample of the pentad in dry MeCN after excitation with 250 fs pulses of 460 nm. The dotted lines from 445–475 and 490–500 nm represent the cutout windows of the laser pulse and the merging of the two measurements. (b) Simplified energy level diagram following excitation of the pentad. After forming the excited photosensitizer, the hole and the electron are initially transferred to the inner D_1_/A_1_ pair, forming a first charge-separated state, abbreviated as CSS-1 with an energy of 1.7 eV above the ground state. Secondary electron transfers then form the final long-lived CSS-2 consisting of the D_2_^•+^/A_2_^•–^ pair with an energy of 1.3 eV. (c) Concentration profile of the three species from (b) obtained by global fitting of the data from (a) to such a three-state model. (d) *Top:* The transient absorption spectrum from (a) with a delay of 100 ps when mainly D_2_–D_1_^•+^–PS–A_1_^•–^–A_2_ is present. *Middle:* The transient absorption spectrum of the reference triad D_1_–PS–A_1_ (see the Supporting Information (SI) for details, Figure S6) immediately after excitation with 10 ns pulses of 470 nm with a signal integration over 200 ns. This TA spectrum represents a direct reference for the expected spectroscopic features of CSS-1. *Bottom:* Difference spectra of PTZ^•+^ (light green) and AQ^•–^ (light violet) obtained after applying potentials that are sufficient to oxidize PTZ to PTZ^•+^ (+1.0 V vs SCE) and to reduce AQ to AQ^•–^ (−1.0 V vs SCE) in suitable reference molecules in dry, Ar-saturated CH_2_Cl_2_ (see Figure S5). The black trace is a linear combination of the two difference spectra that are arbitrarily scaled to compensate for differences in the extinction coefficients. The gray dashed lines mark the individual maxima and serve as guides for the eye. (e) *Top:* The transient absorption spectrum from (a) with a delay of 5000 ps when only D_2_^•+^–D_1_–PS–A_1_–A_2_^•–^ is present. *Middle:* The transient absorption spectrum of a 20 μM solution of the pentad immediately after excitation with 10 ns pulses of 470 nm and an integration of the detection signal over 200 ns. This TA spectrum shows the final charge-separated state CSS-2 that is present on nano- to microsecond time scales. *Bottom:* Difference spectra of TAA^•+^ (dark green) and NDI^•–^ (dark violet) obtained after applying potentials that are sufficient to oxidize TAA to TAA^•+^ (+0.8 V vs SCE) and to reduce NDI to NDI^•–^ (−0.7 V vs SCE) in suitable reference molecules in dry, Ar-saturated CH_2_Cl_2_ (see Figure S5). The black trace is the linear combination of the two difference spectra that are arbitrarily scaled to compensate for differences in extinction coefficients. The gray dashed lines mark the individual maxima and serve as guides for the eye.

This forms the final charge-separated state CSS-2 with an energy of 1.3 eV above the ground state. The relative order of the secondary electron transfers cannot be disentangled, too. It is expected that they both occur on similar time scales as they are very similar in terms of driving forces, distance, and bridge electronics. Disentangling the exact sequence of these individual electron transfer steps is unnecessary for the purposes of our study and is beyond the scope here.

Overall, this enables us to simplify the energy level diagram to three states: the excited photosensitizer *PS, CSS-1, and CSS-2 (Figure 3b). The individual lifetimes of these states can be obtained by globally fitting the data in Figure 3a to the three-state model in Figure 3b. This provides a rate constant for the formation of CSS-1 from the excited state of 7.7 × 10^10^ s^–1^ (Figure 3c). This is on the same time scale as previously shown for similar molecules like the short triad from Figure 1a, where overall charge separation occurs over a comparable distance and with a similar driving force.^68^ CSS-1 then evolves into CSS-2 with a rate constant of 1.0 × 10^9^ s^–1^ (Figure 3c). These secondary electron transfers are comparably slow, despite having very similar driving forces and bridges to overcome. The exact reasons for this behavior remain unclear, but electron transfer rates are generally determined by the interplay of driving force, reorganization energy, and electronic coupling.^87,88^

In our case, the distance between TAA and PTZ^•+^ is relatively short, and the covalent attachment of NDI to the rest of the pentad via its naphthalene core likely facilitates strong electronic coupling.^89^ These factors suggest that the slower formation of CSS-2 compared with CSS-1 is more likely due to differences in reorganization energy rather than electronic coupling.

The transient absorption spectrum of CSS-1 (the 100 ps trace in Figure 3a,d) can be compared to the nanosecond time scale transient absorption spectrum of the reference triad consisting only of D_1_–PS–A_1_ (Figure 3d, top vs middle). The same absorption features can be observed in both spectra, indicating that in both cases, AQ^•–^ and PTZ^•+^ are present. These transient absorption bands can be assigned to the individual molecular components by comparison to electrochemically obtained difference spectra of AQ^•–^ and PTZ^•+^ (Figure 3d, bottom). The peaks at 440 and 600 nm can be assigned to AQ^•–^ and the shoulder at 670 nm, where AQ^•–^ itself does not absorb anymore, to PTZ^•+^. The AQ^•–^ signals do not completely match the reference signals that would suggest absorption at 420 and 560 nm, but the agreement between transient absorption and spectro-electrochemical data remains convincing. Comparison of the transient absorption spectrum after 5000 ps with the one that was time-integrated over the first 200 ns confirms it to be the final, long-lived CSS-2 state (Figure 3e, top vs middle). The individual maxima can be assigned using the difference spectra obtained from electrochemical oxidation of TAA to TAA^•+^ and reduction of NDI to NDI^•–^ (Figure 3e, bottom). The maxima at 480, 600, 690, and 770 nm can be clearly assigned to NDI^•–^. The signal at 390 nm results from the combination of the overlapping bleach of charge neutral NDI with the signal from TAA^•+^. The main feature of TAA^•+^ has a maximum above 900 nm (see Figure S5), and the start of the rise can be seen in the transient absorption spectrum with picosecond resolution (dashed vertical line at the far right of Figure 3e, and rise of the optical density near 900 nm in Figure 3a with increasing delay time).

### A High Quantum Yield of Charge Separation and a Slow Charge Recombination

The decay of the final charge-separated state can be investigated by transient absorption spectroscopy with nanosecond resolution. Upon excitation with 470 nm pulses of ca. 10 ns duration, the absorption features of CSS-2 immediately appear and decay uniformly on a 100 μs time scale (Figure 4a). On this time scale, bimolecular recombination processes are occurring as shown with concentration-dependent experiments, discussed in previous publications.^43,58^ Therefore, we do not observe a single-exponential decay of the transient signals as expected if the intramolecular charge recombination between TAA^•+^ and NDI^•–^ is the sole process. We rather have a time-dependent, multispecies system that requires treatment as a system of coupled differential equations. In a first approximation, we fitted the decay with a simplified triexponential model.^58^ This provided a lifetime of ca. 120 μs for the process changing the least with varying concentrations. Consequently, we attributed this to the inherent intramolecular charge recombination.^58^

**Figure 4:**
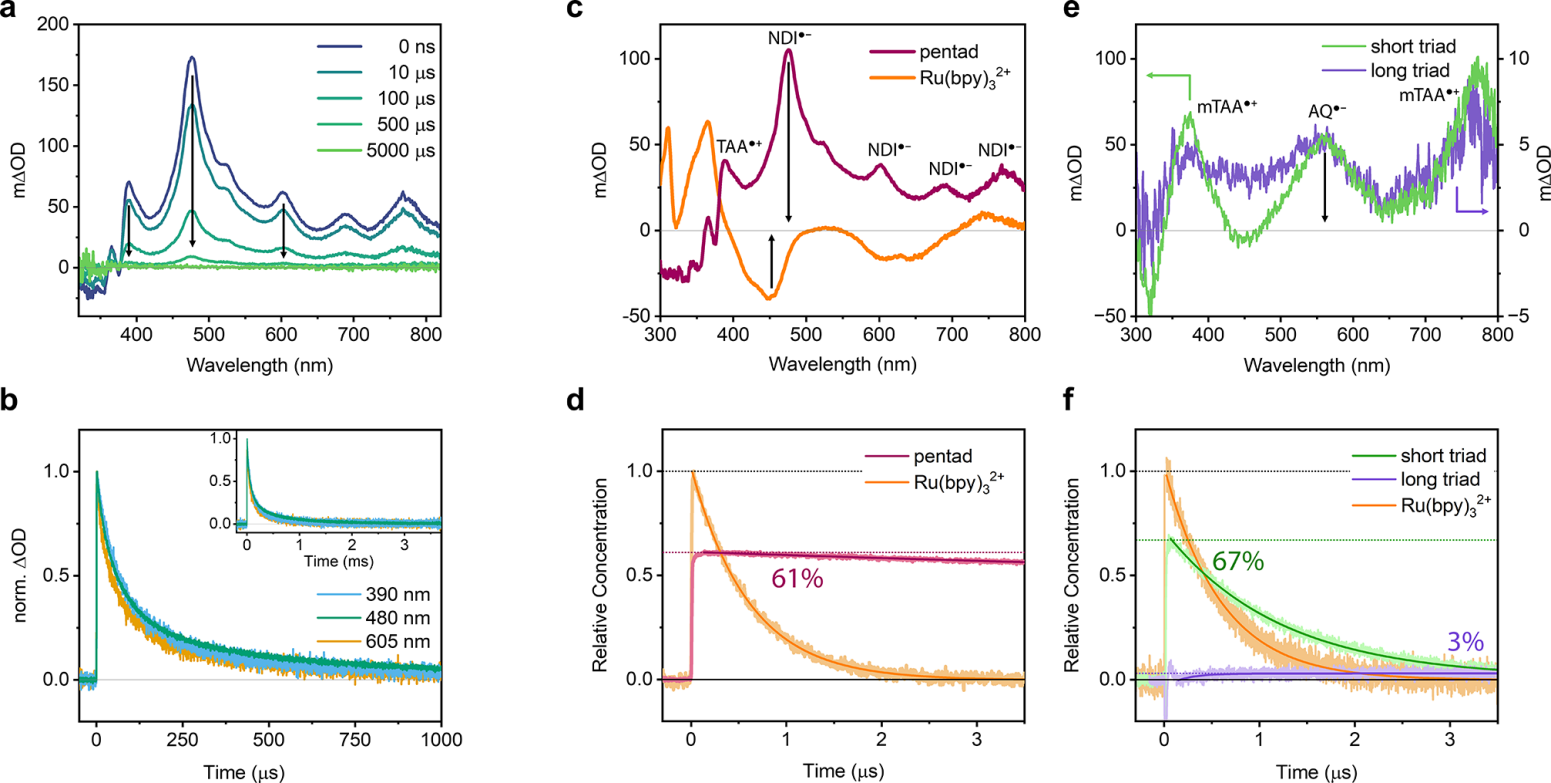
(a) Transient absorption spectra with the indicated delays after exciting a 20 μM solution of the pentad in dry, argon-saturated MeCN at 20 °C with 460 nm laser pulses of ca. 10 ns length. The arrows indicate the three wavelengths that were monitored in (b). (b) Kinetic traces of the (normalized) transient absorption signals at 390 nm (TAA^•+^), and at 480 and 605 nm (NDI^•–^) of a 10 μM solution of the pentad in dry, argon-saturated MeCN after excitation with 460 nm laser pulses of ca. 10 ns length. The inset shows the complete decay of CSS-2 on a ms time scale. (c) Transient absorption spectra of the pentad (purple) in dry, argon-saturated MeCN and Ru­(bpy)_3_Cl_2_ (orange) in argon-saturated H_2_O. Both samples were excited under strictly identical conditions one after another with 460 nm laser pulses of ca. 10 ns duration (ca. 17 mJ), and the signals were time-integrated over the first 200 ns. The black arrows indicate the wavelengths where the decays of CSS-2 in the pentad and the ^3^MLCT excited state of Ru­(bpy)_3_^2+^ were measured in (d). (d) Concentration profiles of the CSS-2 of the pentad and the ^3^MLCT of Ru­(bpy)_3_^2+^ relative to the maximum of the ^3^MLCT excited state. (The bleach recovery signal of Ru­(bpy)_3_^2+^ at 460 nm was multiplied by −1 to obtain the concentration profile and then normalized to a value of 1 at *t* = 0.) The CSS-2 of the pentad reaches 61% of the Ru­(bpy)_3_^2+^ reference signal, corresponding to a charge separation quantum yield of 61%. The finer details of this relative actinometry experiment are explained in Figure S9. (e) Transient absorption spectra of the short triad from Figure 1a (green), time-integrated over 40 ns without a delay, and of the long triad from Figure 1b (violet), time-integrated over 1 μs with a delay of 3 μs. Both triads where excited at 460 nm with pulses of ca. 10 ns duration. At this wavelength, the absorbance of both samples was adjusted to be identical and the laser measurements were conducted under strictly identical conditions, with the two samples measured sequentially, one immediately after the other. The integration times were chosen such that there is negligible photoproduct decay during this time. The delay for the long triad was necessary because of the slow formation of the CSS in this specific case, see main text. The absorption features of AQ^•–^ can be observed at 560 nm while those belonging to monomethoxy-TAA^•+^ (mTAA^•+^) are found at 370 and 770 nm. From this experiment, the quantum yield of charge separation Φ_CSS_ in the short triad is ca. 10-fold higher than that in the long triad. (f) Identical to (d), solutions of the short and the long triad in dry, argon-saturated MeCN and a reference of Ru­(bpy)_3_Cl_2_ in argon-saturated H_2_O with identical absorbance at 460 nm were prepared (see the SI for details, Figure S10). After the excitation of these samples with 460 nm pulses under strictly identical excitation conditions, kinetic traces of the transient absorption signals were measured. After normalization to the ^3^MLCT recovery (treated in the same was as in (d)), concentration profiles of the decay of the CSS in the short triad (green) and in the long triad (violet) and the ^3^MLCT of Ru­(bpy)_3_^2+^ (orange) were obtained. The data for the long triad were emission-corrected to only show the formation of the CSS (see the SI for details, Figure S11). The corresponding quantum yields for CSS formation are 67% (short triad) and 3% (long triad).

The high signal intensity of CSS-2 despite separating the charges by over 44 Å made us curious to investigate the efficiency of the charge separation. Relative actinometry proved to be more precise to investigate the charge separation process than investigations of all relevant reaction rates since dark reactions can be missed by the kinetic treatment.^80^

For relative actinometry experiments, a sample solution and a reference solution with identical optical densities at the excitation wavelength are required. Together with an identical laser excitation power, this ensures the absorption of an essentially identical number of photons. Ru­(bpy)_3_^2+^ has a intersystem crossing quantum yield Φ_ISC_ of 100%, meaning that all absorbed photons contribute to the ^3^MLCT signal, whose change in molecular extinction coefficient at 450 nm (Δε_450 nm_) is known to be –11,000 M^–1^ cm^–1^.^90^ Also, the changes in extinction coefficients of NDI^•–^ (Δε_476 nm_ = 46,600 M^–1^ cm^–1^)^58^ and AQ^•–^ (Δε_560 nm_ = 15,100 M^–1^ cm^–1^)^80^ relative to their redox neutral forms are known. For AQ^•–^, Δε_560 nm_ is assumed to be identical in the short and long triad. With these Δε values in hand, changes in optical density upon excitation can be converted to concentrations relative to the quantitative formation of the ^3^MLCT excited state of Ru­(bpy)_3_^2+^ immediately after the excitation (see the SI for details, Figures S9 and S10).^91^

Solutions of the pentad in dry, argon-saturated MeCN and Ru­(bpy)_3_Cl_2_ in argon-saturated H_2_O were adjusted such that they have the same absorbance at the excitation wavelength (Figure S9a). To ensure identical excitation power, both solutions were excited with 460 nm laser pulses immediately after one another, while keeping all excitation and measurement conditions strictly identical. Kinetic traces of the decay and recovery of the transient signals were measured at the maximum of the NDI^•–^-related absorption (476 nm) for the pentad and at the minimum of the ground-state bleach (455 nm) for the Ru­(bpy)_3_^2+^ reference. With the known Δε values at these wavelengths, the differences in optical density were converted into concentrations and then normalized to the initial ^3^MLCT signal (at *t* = 0) that is formed quantitatively. This allows to obtain the quantum yield for the formation of CSS-2 (Φ_CSS_) relative to the ^3^MLCT-excited Ru­(bpy)_3_^2+^ and was determined to be 61% for the pentad (Figure 4d).

We then aimed to explore the quantum yields of the two representative triads from Figure 1. These were previously treated by an analysis of the rates of the relevant electron transfers for the short triad^68^ or qualitatively from the lower ΔOD of the short triad compared to the long triad.^92^ However, the kinetic analysis yielded an incorrect Φ_CSS_ of 100%, as it failed to account for spontaneous charge recombination following the initial reduction of the photosensitizer and oxidation of the acceptor.^68,93^ We later demonstrated that relative actinometry can reveal significant discrepancies compared to the kinetic method.^80^ To get a more comparable and complete understanding of the remarkable quantum yield for CSS-2 formation in the pentad, we aimed to determine the Φ_CSS_ values for the triads using relative actinometry. This experimental approach offers a direct means of quantifying photoproduct concentrations and quantum yields.

The treatment of the data is explained in detail in Figure S10. For the short triad, a Φ_CSS_ of 67% is obtained for the AQ^•–^-related transient absorption at 560 nm (Figure 4f, green) and 69% for the monomethoxy-TAA^•+^ (mTAA^•+^)-related absorption at 770 nm (Figure S10c). The transient absorption signal of the long triad was corrected for residual emission from the decaying ^3^MLCT excited state during charge separation (Figure S11), yielding a purer CSS-related absorption signal (Figure 4f, violet). However, this is just a cosmetic treatment, since the mΔOD value after the charge separation is complete (after ca. 1.5 μs) is independent of the remaining emission observed at earlier times. For the long triad, a Φ_CSS_ of 3% is obtained for both the AQ^•–^-related transient absorption at 560 nm (Figure 4f, violet) and the mTAA^•+^-related absorption at 770 nm (Figure S10e).

Because of the low signal-to-noise ratio in the data for the long triad, this Φ_CSS_ was also determined via the signal intensity of the time-integrated transient absorption spectra. The spectra from Figure 4e were obtained from exciting solutions of the short and long triads with identical absorbances at the excitation wavelength of 460 nm and with identical laser power. To obtain sufficient quality data with reasonably accurate ΔOD values, the time integration windows for the two samples had to be tailored. For the immediately formed but faster-decaying CSS in the short triad, a measurement window with 0 ns delay and an integration time of 40 ns was used. For the slower formed CSS in the long triad, the initial rise had to be cut out by using a delay of 3 μs and a longer integration window of 1 μs was used to obtain a useful signal-to-noise ratio despite the low quantum efficiency. At 560 nm, the difference in optical density for the short triad is mΔOD = 54 and for the long triad, it is mΔOD = 5.3. Assuming that the AQ^•–^ absorption has the same Δε_560nm_ in both triads, this indicates a relative Φ_CSS_ of 10% of the long triad with respect to the short triad. Together with an Φ_CSS_ of 67% for the short triad (as determined above), this method estimates a Φ_CSS_ of 7% for the long triad. The average of the two experiments in Figure 4e,f is 5%.

The quantum yield for charge separation in the pentad is therefore eight times higher than in the long triad despite the fact that the estimated center-to-center distance between the terminal donor and acceptor units is approximately 5 Å longer in the pentad (Figure 1). In the long triad, formation of the charge-separated state (CSS) involves two long-range electron (or hole) tunneling events: one between the donor and the excited Ru­(bpy)_3_^2+^ photosensitizer and another between the reduced photosensitizer and the acceptor. Both processes occur over distances of roughly 14 Å. Such tunneling events are highly distance dependent due to the exponential decrease of orbital overlap between individual molecular components, as described by the superexchange formalism.^81,94–96^ As a result, electron tunneling rates decrease exponentially with distance, governed by the so-called distance decay constant, β. For the *p*-xylene bridges used in both the pentad and the triads, β values of 1.1 Å^–1^ have been previously reported,^97,98^ significantly higher than for unsubstituted phenyl units, which offer greater π-conjugation and thus lower tunneling barriers. Consequently, charge separation in the long triad becomes much slower and less competitive with energy dissipation pathways. In contrast, the pentad replaces these two long-range tunneling steps with four short-range hopping steps, leading to a significantly higher quantum yield for charge separation.

Importantly, the redox gradient of the pentad is carefully designed to promote hopping only in the direction of photoinduced charge separation but not for the thermal recombination of the final charge-separated state. As a result, energy-storing processes are strongly accelerated and become far more efficient than those in the long triad, whereas the energy-wasting decay of the final CSS remains similarly slow. The intramolecular recombination lifetime of 120 μs in the pentad (and contributions from bimolecular processes extending into the millisecond range) is not a record in absolute terms in purely molecular compounds^46,47,57,99^ or TiO_2_-bound systems.^48,100–102^ However, the key innovation lies in the ability to achieve such a long-lived charge-separated state with a high quantum yield (61%) over a distance approaching 5 nm. To our knowledge, no previous studies have demonstrated this in purely molecular donor–photosensitizer–acceptor compounds.

## Conclusions

In modern synthetic photoredox chemistry, excited-state lifetimes ranging from a few nanoseconds up to microseconds are generally sufficient to support diffusion-based bimolecular reactions, as these typically employ relatively high substrate concentrations and excitation densities.^103–105^ Additionally, many photoredox reactions involve only a single light-dependent step. In contrast, the formation of chemical fuels using sunlight presents more fundamental challenges.^2–4^ The visionary concept of artificial photosynthesis requires multiple light-driven elementary reaction steps and must operate under significantly lower excitation densities.^5,6^ The solar photon flux is inherently low, providing ca. 1100 visible photons per second at the molecular scale relevant to this study (ca. 100 Å^2^).^1,58^ However, the typical rate of photon absorption by molecules under solar irradiance is rather on a seconds time scale.^5,6^ As a result, enabling multiple consecutive light-driven electron transfer steps under such low-light conditions demands that the initial photon absorption efficiently generates a very long-lived charge-separated state.^106^ Without this, mimicking the multielectron transfer processes of the Kok cycle in natural photosynthesis becomes extremely difficult in artificial systems, even with antenna systems designed to enhance the light absorption.^107^ In this work, we have made a critical advance toward enabling the use of low irradiance to achieve charge separation relevant to artificial photosynthesis by demonstrating the molecular design principles that increase the quantum efficiency. Prior studies in this area have primarily focused on reaction kinetics instead of quantum efficiencies, and earlier molecular designs likely could not have supported such efficient charge separation as our molecular pentad now enables.

Long lifetimes of charge-separated states have often been achieved in previous studies by increasing the distance between donor and acceptor units. However, this approach typically compromises the efficiency. In this work, we demonstrate this trade-off by directly comparing two D–PS–A triads, differing in the distance between the electron donor and acceptor, with a pentad in which the terminal donor and acceptor are separated by a similar distance as in the longer triad (Figure 1). Unlike the triads, which rely on passive xylene spacers, the pentad integrates redox-active bridging units that actively mediate electron transfer, enabling not only equivalent but also even greater spatial separation. This experimental design allowed us to make a direct comparison of the absolute quantum yields for photoinduced charge separation via single-step tunneling versus multistep hopping. Although hopping mechanisms have been shown to accelerate electron transfer kinetics in numerous biological and artificial systems,^52–55^ their impact on the quantum yields of charge separation in D–PS–A molecules has, until now, remained largely unexplored.

The relay station strategy implemented in our pentad mimics the function of the multiple redox relay centers found in natural photosynthetic reaction centers.^1,4,108^ Given this analogy, it was to some extent expected that the pentad would exhibit improved quantum yields for charge separation compared with a triad of similar length. However, since previous studies have focused primarily on kinetic measurements, they have not been able to quantitatively determine whether faster light-induced elementary processes actually translate into higher quantum yields. This is because light-independent (dark) charge recombination events often go undetected.^68,80,93^ The eightfold increase in quantum yield observed for the pentad relative to the triad is therefore striking, and the absolute quantum yield of 61% achieved by the pentad is unexpectedly high. Nevertheless, despite comparable separation distance and similar photochemical energy conversion efficiency, the intrinsic quantum yields for charge separation observed in natural photosynthesis (typically close to unity) remain unmatched.^1,4,108,109^

In conclusion, the molecular design strategy presented here enables long-lived charge separation across substantial distances without significantly compromising the charge-separation efficiency. We believe that this is an important conceptual advance for the design of future solar energy conversion systems.

## Supplementary Material



## Notes

General procedures and equipment details and (spectro)­electrochemical and transient absorption spectroscopy data (PDF)

## References

[1] Blankenship, R. E. Molecular Mechanisms of Photosynthesis, 2. ed.; Wiley Blackwell: Chichester, 2014.

[2] Gust D., Moore T. A., Moore A. L. (2009). Solar Fuels
via Artificial Photosynthesis. Acc. Chem. Res..

[3] Kornienko N., Zhang J. Z., Sakimoto K. K., Yang P., Reisner E. (2018). Interfacing
nature’s catalytic machinery with synthetic materials for semi-artificial
photosynthesis. Nat. Nanotechnol..

[4] Proppe A. H., Li Y. C., Aspuru-Guzik A., Berlinguette C. P., Chang C. J., Cogdell R., Doyle A. G., Flick J., Gabor N. M., Van Grondelle R., Hammes-Schiffer S., Jaffer S. A., Kelley S. O., Leclerc M., Leo K., Mallouk T. E., Narang P., Schlau-Cohen G. S., Scholes G. D., Vojvodic A., Yam V. W.-W., Yang J. Y., Sargent E. H. (2020). Bioinspiration in light harvesting and catalysis. Nat. Rev. Mater..

[5] Hammarström L. (2015). Accumulative
Charge Separation for Solar Fuels Production: Coupling Light-Induced
Single Electron Transfer to Multielectron Catalysis. Acc. Chem. Res..

[6] Pellegrin Y., Odobel F. (2011). Molecular devices featuring
sequential photoinduced
charge separations for the storage of multiple redox equivalents. Coord. Chem. Rev..

[7] Gotico P., Tran T., Baron A., Vauzeilles B., Lefumeux C., Ha-Thi M., Pino T., Halime Z., Quaranta A., Leibl W., Aukauloo A. (2021). Tracking Charge
Accumulation
in a Functional Triazole-Linked Ruthenium-Rhenium Dyad Towards Photocatalytic
Carbon Dioxide Reduction. ChemPhotoChem..

[8] Matt B., Fize J., Moussa J., Amouri H., Pereira A., Artero V., Izzet G., Proust A. (2013). Charge photo-accumulation
and photocatalytic hydrogen evolution under visible light at an iridium­(iii)-photosensitized polyoxotungstate. Energy Environ. Sci..

[9] Appel A. M., Bercaw J. E., Bocarsly A. B., Dobbek H., DuBois D. L., Dupuis M., Ferry J. G., Fujita E., Hille R., Kenis P. J. A., Kerfeld C. A., Morris R. H., Peden C. H. F., Portis A. R., Ragsdale S. W., Rauchfuss T. B., Reek J. N. H., Seefeldt L. C., Thauer R. K., Waldrop G. L. (2013). Frontiers,
Opportunities, and Challenges in Biochemical and Chemical Catalysis
of CO_2_ Fixation. Chem. Rev..

[10] Berardi S., Drouet S., Francàs L., Gimbert-Suriñach C., Guttentag M., Richmond C., Stoll T., Llobet A. (2014). Molecular
artificial photosynthesis. Chem. Soc. Rev..

[11] Wagner A., Sahm C. D., Reisner E. (2020). Towards molecular
understanding of
local chemical environment effects in electro- and photocatalytic
CO_2_ reduction. Nat. Catal..

[12] Schulz M., Hagmeyer N., Wehmeyer F., Lowe G., Rosenkranz M., Seidler B., Popov A., Streb C., Vos J. G., Dietzek B. (2020). Photoinduced Charge Accumulation and Prolonged Multielectron
Storage for the Separation of Light and Dark Reaction. J. Am. Chem. Soc..

[13] Arias-Rotondo D. M., McCusker J. K. (2016). The photophysics
of photoredox catalysis: a roadmap
for catalyst design. Chem. Soc. Rev..

[14] Sakizadeh J. D., Weiss R., Scholes G. D., Kudisch B. (2025). Ultrafast Spectroscopy
and Dynamics of Photoredox Catalysis. Annu.
Rev. Phys. Chem..

[15] Chen X., Zhang X., Xiao X., Wang Z., Zhao J. (2023). Recent Developments
on Understanding Charge Transfer in Molecular Electron Donor-Acceptor
Systems. Angew. Chem., Int. Ed..

[16] Strahan J., Popere B. C., Khomein P., Pointer C. A., Martin S. M., Oldacre A. N., Thayumanavan S., Young E. R. (2019). Modulating absorption
and charge transfer in bodipy-carbazole donor–acceptor dyads
through molecular design. Dalton Trans..

[17] Harvey S.
M., Wasielewski M. R. (2021). Photogenerated
Spin-Correlated Radical Pairs: From
Photosynthetic Energy Transduction to Quantum Information Science. J. Am. Chem. Soc..

[18] Mims D., Herpich J., Lukzen N. N., Steiner U. E., Lambert C. (2021). Readout of
spin quantum beats in a charge-separated radical pair by pump-push
spectroscopy. Science.

[19] Eckvahl H. J., Tcyrulnikov N. A., Chiesa A., Bradley J. M., Young R. M., Carretta S., Krzyaniak M. D., Wasielewski M. R. (2023). Direct
observation of chirality-induced spin selectivity in electron donor–acceptor
molecules. Science.

[20] Hu G., Kang H. S., Mandal A. K., Roy A., Kirmaier C., Bocian D. F., Holten D., Lindsey J. S. (2018). Synthesis
of arrays
containing porphyrin, chlorin, and perylene-imide constituents for
panchromatic light-harvesting and charge separation. RSC Adv..

[21] Wolf M., Herrmann A., Hirsch A., Guldi D. M. (2017). Rigid, Branched
Porphyrin Antennas: Control over Cascades of Unidirectional Energy
Funneling and Charge Transfer. J. Am. Chem.
Soc..

[22] Poddutoori P. K., Kandrashkin Y. E., Obondi C. O., D’Souza F., Van Der Est A. (2018). Triplet electron transfer and spin polarization in
a palladium porphyrin–fullerene conjugate. Phys. Chem. Chem. Phys..

[23] Kaur R., Possanza F., Limosani F., Bauroth S., Zanoni R., Clark T., Arrigoni G., Tagliatesta P., Guldi D. M. (2020). Understanding and Controlling Short-
and Long-Range
Electron/Charge-Transfer Processes in Electron Donor-Acceptor Conjugates. J. Am. Chem. Soc..

[24] Yadagiri B., Kaswan R. R., Tagare J., Kumar V., Rajesh M. N., Singh S. P., Karr P. A., D’Souza F., Giribabu L. (2024). Excited Charge Separation in a π-Interacting
Phenothiazine-Zinc Porphyrin–Fullerene Donor-Acceptor Conjugate. J. Phys. Chem. A.

[25] Zarrabi N., Obondi C. O., Lim G. N., Seetharaman S., Boe B. G., D’Souza F., Poddutoori P. K. (2018). Charge-separation
in panchromatic, vertically positioned bis­(donor styryl)­BODIPY–aluminum­(iii) porphyrin–fullerene supramolecular triads. Nanoscale.

[26] Zarrabi N., Poddutoori P. K. (2021). Aluminum­(III)
porphyrin: A unique building block for
artificial photosynthetic systems. Coord. Chem.
Rev..

[27] Wasielewski M. R. (1992). Photoinduced
electron transfer in supramolecular systems for artificial photosynthesis. Chem. Rev..

[28] Bagemihl B., Pannwitz A., Rau S. (2023). Gatekeeping Effect of Ancillary Ligand
on Electron Transfer in Click Chemistry-Linked Tris-Heteroleptic Ruthenium­(II)
Donor–Photosensitizer–Acceptor Triads. Solar RRL.

[29] Luo Y., Barthelmes K., Wächtler M., Winter A., Schubert U. S., Dietzek B. (2017). Increased
Charge Separation Rates with Increasing Donor-Acceptor
Distance in Molecular Triads: The Effect of Solvent Polarity. J. Phys. Chem. C.

[30] Arrigo A., Nastasi F., La Ganga G., Puntoriero F., Zappalà G., Licciardello A., Cavazzini M., Quici S., Campagna S. (2017). Solvent-control of
photoinduced electron
transfer via hydrogen bonding in a molecular triad made of a dinuclear
chromophore subunit. Chem. Phys. Lett..

[31] Sebastian E., Hariharan M. (2023). A Symmetry-Broken
Charge-Separated State in the Marcus
Inverted Region. Angew. Chem., Int. Ed..

[32] Miura T., Fujiwara D., Akiyama K., Horikoshi T., Suzuki S., Kozaki M., Okada K., Ikoma T. (2017). Magnetic Control
of the Charge-Separated State Lifetime Realized by Covalent Attachment
of a Platinum Complex. J. Phys. Chem. Lett..

[33] Klein J. H., Schmidt D., Steiner U. E., Lambert C. (2015). Complete Monitoring
of Coherent and Incoherent Spin Flip Domains in the Recombination
of Charge-Separated States of Donor-Iridium Complex-Acceptor Triads. J. Am. Chem. Soc..

[34] Sukegawa J., Schubert C., Zhu X., Tsuji H., Guldi D. M., Nakamura E. (2014). Electron transfer through
rigid organic molecular wires
enhanced by electronic and electron–vibration coupling. Nat. Chem..

[35] Delor M., Scattergood P. A., Sazanovich I. V., Parker A. W., Greetham G. M., Meijer A. J. H. M., Towrie M., Weinstein J. A. (2014). Toward
control of electron transfer in donor-acceptor molecules by bond-specific
infrared excitation. Science.

[36] Delor M., Archer S. A., Keane T., Meijer A. J. H. M., Sazanovich I. V., Greetham G. M., Towrie M., Weinstein J. A. (2017). Directing
the path of light-induced electron transfer at a molecular fork using
vibrational excitation. Nat. Chem..

[37] Pfeffer M.
G., Müller C., Kastl E. T. E., Mengele A. K., Bagemihl B., Fauth S. S., Habermehl J., Petermann L., Wächtler M., Schulz M., Chartrand D., Laverdière F., Seeber P., Kupfer S., Gräfe S., Hanan G. S., Vos J. G., Dietzek-Ivanšić B., Rau S. (2022). Active repair of a dinuclear photocatalyst for visible-light-driven
hydrogen production. Nat. Chem..

[38] Suarez-Antuna I., Lalaoui N., Chaigne-Tarlotin E., Molton F., Loiseau F., Duboc C. (2025). Unraveling Photo-Driven
H_2_ Production with a Bio-Inspired
NiFe Complex: Revealing an Unexpected Hydrogen Atom Source. J. Am. Chem. Soc..

[39] Bürgin T. H., Wenger O. S. (2021). Recent Advances and Perspectives in Photodriven Charge
Accumulation in Molecular Compounds: A Mini Review. Energy Fuels.

[40] Fukuzumi S., Ohkubo K., Suenobu T. (2014). Long-Lived
Charge Separation and
Applications in Artificial Photosynthesis. Acc.
Chem. Res..

[41] Karlsson S., Boixel J., Pellegrin Y., Blart E., Becker H.-C., Odobel F., Hammarström L. (2010). Accumulative Charge Separation Inspired
by Photosynthesis. J. Am. Chem. Soc..

[42] Favereau L., Makhal A., Pellegrin Y., Blart E., Petersson J., Göransson E., Hammarström L., Odobel F. (2016). A Molecular Tetrad
That Generates a High-Energy Charge-Separated State by Mimicking the
Photosynthetic Z-Scheme. J. Am. Chem. Soc..

[43] Kuss-Petermann M., Wenger O. S. (2016). Electron Transfer Rate Maxima at Large Donor-Acceptor
Distances. J. Am. Chem. Soc..

[44] Kleine A., Mankel C., Hainthaler A., Wächtler M., Dietzek-Ivanšić B., Schubert U. S., Jäger M. (2024). From Molecular
to Polymeric Donors: Prolonged Charge Separation in Modular Photoredox-Active
Ru­(II) Polypyridyl-Type Triads. Inorg. Chem..

[45] Wei Z., Philip A. M., Jager W. F., Grozema F. C. (2022). Fast Charge Separation
in Distant Donor-Acceptor Dyads Driven by Relaxation of a Hot Excited
State. J. Phys. Chem. C.

[46] Imahori H., Guldi D. M., Tamaki K., Yoshida Y., Luo C., Sakata Y., Fukuzumi S. (2001). Charge Separation
in a Novel Artificial
Photosynthetic Reaction Center Lives 380 ms. J. Am. Chem. Soc..

[47] Borgström M., Shaikh N., Johansson O., Anderlund M. F., Styring S., Åkermark B., Magnuson A., Hammarström L. (2005). Light Induced
Manganese Oxidation and Long-Lived Charge Separation in a Mn_2_
^II,II^–Ru^II^(bpy)_3_–Acceptor
Triad. J. Am. Chem. Soc..

[48] Sampaio R. N., Troian-Gautier L., Meyer G. J. (2018). A Charge-Separated State that Lives
for Almost a Second at a Conductive Metal Oxide Interface. Angew. Chem., Int. Ed..

[49] Wolf M., Costa J. I. T., Minameyer M. B., Drewello T., Tomé A. C., Guldi D. M. (2019). Efficient Low Driving
Force Charge Separation in an
Electron Deficient Zn-Porphyrin–Fullerene Donor-Acceptor Conjugate. J. Phys. Chem. C.

[50] Megiatto J. D., Méndez-Hernández D. D., Tejeda-Ferrari M. E., Teillout A.-L., Llansola-Portolés M. J., Kodis G., Poluektov O. G., Rajh T., Mujica V., Groy T. L., Gust D., Moore T. A., Moore A. L. (2014). A bioinspired
redox relay that mimics radical interactions of the Tyr–His
pairs of photosystem II. Nat. Chem..

[51] Odella E., Mora S. J., Wadsworth B. L., Huynh M. T., Goings J. J., Liddell P. A., Groy T. L., Gervaldo M., Sereno L. E., Gust D., Moore T. A., Moore G. F., Hammes-Schiffer S., Moore A. L. (2018). Controlling Proton-Coupled
Electron Transfer in Bioinspired
Artificial Photosynthetic Relays. J. Am. Chem.
Soc..

[52] Shih C., Museth A. K., Abrahamsson M., Blanco-Rodriguez A. M., Di Bilio A. J., Sudhamsu J., Crane B. R., Ronayne K. L., Towrie M., Vlček A., Richards J. H., Winkler J. R., Gray H. B. (2008). Tryptophan-Accelerated Electron Flow Through Proteins. Science.

[53] Takematsu K., Williamson H., Blanco-Rodríguez A. M., Sokolová L., Nikolovski P., Kaiser J. T., Towrie M., Clark I. P., Vlček A., Winkler J. R., Gray H. B. (2013). Tryptophan-Accelerated
Electron Flow Across a Protein-Protein Interface. J. Am. Chem. Soc..

[54] Cordes M., Giese B. (2009). Electron transfer in
peptides and proteins. Chem. Soc. Rev..

[55] Natali M., Campagna S., Scandola F. (2014). Photoinduced
electron transfer across
molecular bridges: electron- and hole-transfer superexchange pathways. Chem. Soc. Rev..

[56] Gust D., Moore T. A., Moore A. L., Barrett D., Harding L. O., Makings L. R., Liddell P. A., De Schryver F. C., Van der Auweraer M. (1988). Photoinitiated charge
separation in a
carotenoid-porphyrin-diquinone tetrad: enhanced quantum yields via
multistep electron transfers. J. Am. Chem. Soc..

[57] Guldi D. M., Imahori H., Tamaki K., Kashiwagi Y., Yamada H., Sakata Y., Fukuzumi S. (2004). A Molecular
Tetrad
Allowing Efficient Energy Storage for 1.6 s at 163 K. J. Phys. Chem. A.

[58] Brändlin M., Pfund B., Wenger O. S. (2025). Photoinduced
Double Charge Accumulation
in a Molecular Compound. Nat. Chem..

[59] Luo C., Guldi D. M., Imahori H., Tamaki K., Sakata Y. (2000). Sequential
Energy and Electron Transfer in an Artificial Reaction Center: Formation
of a Long-Lived Charge-Separated State. J. Am.
Chem. Soc..

[60] Bottari G., De La Torre G., Guldi D. M., Torres T. (2010). Covalent and Noncovalent
Phthalocyanine–Carbon Nanostructure Systems: Synthesis, Photoinduced
Electron Transfer, and Application to Molecular Photovoltaics. Chem. Rev..

[61] KC C. B., D’Souza F. (2016). Design and
photochemical study of supramolecular donor–acceptor
systems assembled via metal–ligand axial coordination. Coord. Chem. Rev..

[62] Barigelletti F., Flamigni L., Balzani V., Collin J.-P., Sauvage J.-P., Sour A., Constable E. C., Thompson A. M. W. C. (1994). Rigid Rod-Like
Dinuclear Ru­(II)/Os­(II) Terpyridine-Type Complexes. Electrochemical
Behavior, Absorption Spectra, Luminescence Properties, and Electronic
Energy Transfer through Phenylene Bridges. J.
Am. Chem. Soc..

[63] Puntoriero F., Sartorel A., Orlandi M., La Ganga G., Serroni S., Bonchio M., Scandola F., Campagna S. (2011). Photoinduced water
oxidation using dendrimeric Ru­(II) complexes as photosensitizers. Coord. Chem. Rev..

[64] Lefebvre J.-F., Schindler J., Traber P., Zhang Y., Kupfer S., Gräfe S., Baussanne I., Demeunynck M., Mouesca J.-M., Gambarelli S., Artero V., Dietzek B., Chavarot-Kerlidou M. (2018). An artificial
photosynthetic system for photoaccumulation
of two electrons on a fused dipyridophenazine (dppz)–pyridoquinolinone
ligand. Chem. Sci..

[65] Mede T., Jäger M., Schubert U. S. (2018). “Chemistry-on-the-complex”:
functional Ru^II^ polypyridyl-type sensitizers as divergent
building blocks. Chem. Soc. Rev..

[66] Sayre H., Ripberger H. H., Odella E., Zieleniewska A., Heredia D. A., Rumbles G., Scholes G. D., Moore T. A., Moore A. L., Knowles R. R. (2021). PCET-Based
Ligand Limits Charge Recombination
with an Ir­(III) Photoredox Catalyst. J. Am.
Chem. Soc..

[67] Xie Z.-L., Gupta N., Niklas J., Poluektov O. G., Lynch V. M., Glusac K. D., Mulfort K. L. (2023). Photochemical charge
accumulation in a heteroleptic copper­(i)-anthraquinone molecular
dyad *via* proton-coupled electron transfer. Chem. Sci..

[68] Hankache J., Niemi M., Lemmetyinen H., Wenger O. S. (2012). Photoinduced Electron
Transfer in Linear Triarylamine–Photosensitizer–Anthraquinone
Triads with Ruthenium­(II), Osmium­(II), and Iridium­(III). Inorg. Chem..

[69] Knoll S., Zens C., Maisuradze T., Schmidt H., Kupfer S., Zedler L., Dietzek-Ivanšić B., Streb C. (2024). Light-Induced Charge Separation in Covalently Linked BODIPY-Quinone-Alkyne
Dyads. Chem. - Eur. J..

[70] Mendes
Marinho S., Ha-Thi M., Pham V., Quaranta A., Pino T., Lefumeux C., Chamaillé T., Leibl W., Aukauloo A. (2017). Time-Resolved Interception of Multiple-Charge
Accumulation in a Sensitizer-Acceptor Dyad. Angew. Chem., Int. Ed..

[71] Chaignon F., Falkenström M., Karlsson S., Blart E., Odobel F., Hammarström L. (2007). Very large
acceleration of the photoinduced electron
transfer in a Ru­(bpy)_3_ – naphthalene bisimide dyad
bridged on the naphthyl core. Chem. Commun..

[72] Bürgin T. H., Ogawa T., Wenger O. S. (2023). Better
Covalent Connection in a Molecular
Triad Enables More Efficient Photochemical Energy Storage. Inorg. Chem..

[73] Weiss E. A., Ahrens M. J., Sinks L. E., Gusev A. V., Ratner M. A., Wasielewski M. R. (2004). Making
a Molecular Wire: Charge and Spin Transport
through *para*-Phenylene Oligomers. J. Am. Chem. Soc..

[74] Barthelmes K., Winter A., Schubert U. S. (2016). Dyads and
Triads Based on Phenothiazine,
Bis­(terpyridine)­ruthenium­(II) Complexes, and Fullerene. Eur. J. Inorg. Chem..

[75] Skaisgirski M., Larsen C. B., Kerzig C., Wenger O. S. (2019). Stepwise Photoinduced
Electron Transfer in a Tetrathiafulvalene-Phenothiazine-Ruthenium
Triad. Chem. - Eur. J..

[76] KC C. B., Lim G. N., Nesterov V. N., Karr P. A., D'Souza F. (2014). Phenothiazine–BODIPY–Fullerene
Triads as Photosynthetic Reaction Center Models: Substitution and
Solvent Polarity Effects on Photoinduced Charge Separation and Recombination. Chem. - Eur. J..

[77] Poddutoori P. K., Sandanayaka A. S. D., Zarrabi N., Hasobe T., Ito O., Van Der Est A. (2011). Sequential Charge Separation in Two Axially Linked
Phenothiazine–Aluminum­(III) Porphyrin–Fullerene Triads. J. Phys. Chem. A.

[78] Speck F., Rombach D., Wagenknecht H.-A. (2019). N-Arylphenothiazines
as strong donors
for photoredox catalysis – pushing the frontiers of nucleophilic
addition of alcohols to alkenes. Beilstein J.
Org. Chem..

[79] Revoju S., Matuhina A., Canil L., Salonen H., Hiltunen A., Abate A., Vivo P. (2020). Structure-induced
optoelectronic
properties of phenothiazine-based materials. J. Mater. Chem. C.

[80] Neumann S., Kerzig C., Wenger O. S. (2019). Quantitative
insights into charge-separated
states from one- and two-pulse laser experiments relevant for artificial
photosynthesis. Chem. Sci..

[81] Wenger O. S. (2011). How Donor–Bridge–Acceptor
Energetics Influence Electron Tunneling Dynamics and Their Distance
Dependences. Acc. Chem. Res..

[82] Walther M. E., Wenger O. S. (2009). Tuning the Rates
of Long-Range Charge Transfer across
Phenylene Wires. ChemPhysChem.

[83] Eng M. P., Albinsson B. (2009). The dependence
of the electronic coupling on energy
gap and bridge conformation – Towards prediction of the distance
dependence of electron transfer reactions. Chem.
Phys..

[84] Das A., Ghosh S. (2016). H-bonding directed
programmed supramolecular assembly of naphthalene-diimide
(NDI) derivatives. Chem. Commun..

[85] Prier C. K., Rankic D. A., MacMillan D. W. C. (2013). Visible
Light Photoredox Catalysis
with Transition Metal Complexes: Applications in Organic Synthesis. Chem. Rev..

[86] Kim H., Keller B., Ho-Wu R., Abeyasinghe N., Vázquez R. J., Goodson T., Zimmerman P. M. (2018). Enacting
Two-Electron Transfer from a Double-Triplet State of Intramolecular
Singlet Fission. J. Am. Chem. Soc..

[87] Barbara P. F., Meyer T. J., Ratner M. A. (1996). Contemporary Issues in Electron Transfer
Research. J. Phys. Chem..

[88] Piechota E. J., Meyer G. J. (2019). Introduction to
Electron Transfer: Theoretical Foundations
and Pedagogical Examples. J. Chem. Educ..

[89] Fortage J., Göransson E., Blart E., Becker H.-C., Hammarström L., Odobel F. (2007). Strongly coupled zinc phthalocyanine–tin porphyrin
dyad performing ultra-fast single step charge separation over a 34
Å distance. Chem. Commun..

[90] Müller P., Brettel K. (2012). [Ru­(bpy)_3_]^2+^ as a reference in
transient absorption spectroscopy: differential absorption coefficients
for formation of the long-lived ^3^MLCT excited state. Photochem. Photobiol. Sci..

[91] Damrauer N. H., Cerullo G., Yeh A., Boussie T. R., Shank C. V., McCusker J. K. (1997). Femtosecond Dynamics
of Excited-State Evolution in
[Ru­(bpy)_3_]^2+^. Science.

[92] Kuss-Petermann M., Wenger O. S. (2016). Increasing Electron-Transfer Rates with Increasing
Donor-Acceptor Distance. Angew. Chem., Int.
Ed..

[93] Hankache J., Wenger O. S. (2011). Microsecond charge recombination in a linear triarylamine–Ru­(bpy)_3_
^2+^–anthraquinone triad. Chem. Commun..

[94] McConnell H. M. (1961). Intramolecular
Charge Transfer in Aromatic Free Radicals. J.
Chem. Phys..

[95] Winkler J. R., Gray H. B. (2014). Long-Range Electron Tunneling. J. Am. Chem. Soc..

[96] Gilbert M., Albinsson B. (2015). Photoinduced
charge and energy transfer in molecular
wires. Chem. Soc. Rev..

[97] Hanss D., Walther M. E., Wenger O. S. (2010). Importance
of covalence, conformational
effects and tunneling-barrier heights for long-range electron transfer:
Insights from dyads with oligo-p-phenylene, oligo-p-xylene and oligo-p-dimethoxybenzene
bridges. Coord. Chem. Rev..

[98] Arrigo A., Santoro A., Puntoriero F., Lainé P. P., Campagna S. (2015). Photoinduced electron transfer in donor–bridge–acceptor
assemblies: The case of Os­(II)-bis­(terpyridine)-(bi)­pyridinium dyads. Coord. Chem. Rev..

[99] Flamigni L., Baranoff E., Collin J., Sauvage J. (2006). A Triad Based
on an
Iridium­(III) Bisterpyridine Complex Leading to a Charge-Separated
State with a 120-μs Lifetime at Room Temperature. Chem. - Eur. J..

[100] Argazzi R., Bignozzi C. A., Heimer T. A., Castellano F. N., Meyer G. J. (1995). Long-Lived Photoinduced Charge Separation
across Nanocrystalline
TiO_2_ Interfaces. J. Am. Chem. Soc..

[101] Hirata N., Lagref J., Palomares E. J., Durrant J. R., Nazeeruddin M. K., Grätzel M., Di Censo D. (2004). Supramolecular Control of Charge-Transfer
Dynamics
on Dye-sensitized Nanocrystalline TiO_2_ Films. Chem. - Eur. J..

[102] McCall K. L., Morandeira A., Durrant J., Yellowlees L. J., Robertson N. (2010). Characterisation
of a ruthenium bipyridyl dye showing
a long-lived charge-separated state on TiO_2_ in the presence
of I^–^/I_3_
^–^. Dalton Trans..

[103] Yoon T. P., Ischay M. A., Du J. (2010). Visible light
photocatalysis
as a greener approach to photochemical synthesis. Nat. Chem..

[104] Marzo L., Pagire S. K., Reiser O., König B. (2018). Visible-Light
Photocatalysis: Does It Make a Difference in Organic Synthesis?. Angew. Chem., Int. Ed..

[105] Talbott E. D., Burnett N. L., Swierk J. R. (2023). Mechanistic
and
kinetic studies of visible light photoredox reactions. Chem. Phys. Rev..

[106] Godin R., Kafizas A., Durrant J. R. (2017). Electron
transfer
dynamics in fuel producing photosystems. Curr.
Opin. Electrochem..

[107] Wang D., Sampaio R. N., Troian-Gautier L., Marquard S. L., Farnum B. H., Sherman B. D., Sheridan M. V., Dares C. J., Meyer G. J., Meyer T. J. (2019). Molecular Photoelectrode
for Water Oxidation Inspired by Photosystem II. J. Am. Chem. Soc..

[108] Durrant J. R. (2013). Molecular approaches to solar energy
conversion: the
energetic cost of charge separation from molecular-excited states. Philos. Trans. R. Soc. A.

[109] Blankenship R. E., Tiede D. M., Barber J., Brudvig G. W., Fleming G., Ghirardi M., Gunner M. R., Junge W., Kramer D. M., Melis A., Moore T. A., Moser C. C., Nocera D. G., Nozik A. J., Ort D. R., Parson W. W., Prince R. C., Sayre R. T. (2011). Comparing Photosynthetic and Photovoltaic
Efficiencies and Recognizing the Potential for Improvement. Science.

