# Supplementary material for: Molecular Design Principles for Achieving High-Efficiency Light-Induced Charge Separation at the Nanometer Scale
Source: JACS Au. 2025 Oct 10;5(10):5157–69. doi: 10.1021/jacsau.5c01092 (PMC12569672; doi:10.1021/jacsau.5c01092)
Supplement: Supplementary file 1 [file au5c01092_si_001.pdf]

Supporting Information for

Molecular Design Principles for Achieving High-Efficiency Light-Induced Charge Separation at the Nanometer Scale

Mathis Brändlin, Felix A. Himmelreich, Oliver S. Wenger*

Department of Chemistry, University of Basel, St. Johanns-Ring 19, 4056 Basel, Switzerland

*Corresponding author: oliver.wenger@unibas.ch

Table of Contents

Materials and Methods 2

Reference Compounds 4

UV-Vis Absorption Spectroscopy 4

Cyclic Voltammetry 5

Energy Level Diagram 6

Spectroelectrochemistry 7

Transient Absorption Spectroscopy: Reference Triads 8

- Reference for the Inner Triad (= D_1-PS-A_1): 8
- Short Triad: 9
- Long Triad: 10

Relative Actinometry: Pentad 12

Relative Actinometry: Short Triad and Long Triad 13

Correction for Luminescence 15

DFT 16

References 18

Materials and Methods

Synthetic procedures for the pentad (and related reference compounds),[1] the short triad[2] and the long triad[3] are already described.

Steady-state UV-vis absorption spectra were recorded using a Cary5000 spectrophotometer from Varian. Samples were prepared in dry solvents under ambient conditions and measured in quartz cuvettes (10 × 10 mm), unless stated otherwise.

Cyclic voltammetry was performed under N_2 or Ar atmosphere in a three-necked electrochemical cell. A glassy carbon disc electrode, a silver wire, and a saturated calomel electrode were used as working, counter, and reference electrodes, respectively. Sample solutions (1.0 mM) were prepared in dry CH_2Cl_2 or CH_3CN with supporting electrolyte (0.1 M, tetra-*n*-butylammonium hexafluorophosphate, $TBAPF_6$) and solutions were bubbled with N_2 or Ar for 5 min prior to each measurement. The scan rate was 100 mV s^{-1} unless stated otherwise and the response current signals were recorded on a Versastat4-200 potentiostat from Princeton Applied Research.

Spectro-electrochemical measurements (SEC) by recording the UV-vis absorbance spectral changes upon electrochemical reduction or oxidation were measured using N_2 bubbled samples in dry CH_2Cl_2 in a cuvette with an optical path length of 1 mm. To a sample solution (typically 250 µM) containing a supporting electrolyte (0.1 M, $TBAPF_6$), continuous voltage was applied using Pt mesh, Pt wire, and saturated calomel electrodes as working, counter, and reference electrodes, respectively. The applied voltage was controlled by a Versastat4-200 potentiostat from Princeton Applied Research. The resulting UV-vis absorbance differences were recorded on a OceanHDX miniature spectrometer from Ocean Optics.

Sample preparation for spectrophotometric measurements were performed using quartz cuvettes with screw septum caps. Solutions were purged with argon for 10 min to remove oxygen.

Transient UV-Vis absorption spectroscopy with nanosecond time resolution was performed on an LP920-KS apparatus from Edinburgh Instruments. A frequency-tripled Nd:YAG laser (Quantel Brilliant, ca. 10 ns pulse width and a pulse frequency of 10 Hz) equipped with an OPO from Opotek was used for excitation at 460-470 nm with a pulse energy of ca. 15 mJ. An iCCD camera from Andor was used to detect transient absorption spectra and single-wavelength kinetics were recorded with a photomultiplier

tube. All measurements were conducted using a temperature-controlled sample holder maintained at 20 °C.

Transient UV-Vis absorption spectroscopy with picosecond time resolution was measured using a commercial pump-probe instrument from Light Conversion using a HARPIA spectrometer and a Kymera 193i-B2 iCCD camera from ANDOR. In this experimental setup, the excitation light is generated by a PHAROS laser (Light Conversion, Yb:KGW laser, source wavelength = 1030 nm, pulse duration = ca. 190 fs, repetition rate = 50 kHz, output power = 10 W, pulse energy = 0.2 mJ), and the actual pump light was generated by an optical parametric amplifier called ORPHEUS (Light Conversion, using ca. 90% of the fundamental pulse). Probe pulses for the spectral range of 500−900 nm were generated using the second optical harmonic of PHAROS (515 nm) and a 5 mm thick sapphire white light supercontinuum generator (WLSc, ca. 10% of the fundamental pulse were used to generate a white light super-continuum). Sample solutions (typically 0.3 mM) were measured in a 1 mm quartz cuvette at room temperature under air. The obtained femtosecond transient absorption datasets were chirp- and background-corrected, and analyzed using the CarpetView software provided by Light Conversion.

Abbreviations used are: xy = *p*-xylene, hxy = 1,4-di(*n*-hexyl)benzene, TAA = *N,N*-bis(3,4-dimethoxyphenyl)-2,5-dimethylaniline, mTAA = monomethoxy triarylamine, *N,N*-bis(4-methoxyphenyl)-2,5-dimethylaniline, PTZ = 10-methyl-10*H*-phenothiazine, bpy = 2,2'-bipyridine, AQ = 9,10-anthraquinone, NDI = naphthalene diimide, 2,7-bis(2-ethylhexyl)benzo[lmn][3,8]phenanthroline-1,3,6,8(2*H*,7*H*)-tetraone, TMS = trimethyl-silyl, NBS = *N*-bromosuccinimide, B_2pin_2 = bis(pinacolato)diboron, dba = di-benzylideneacetone, dppf = 1,1'-bis(diphenylphosphino)ferrocene, XPhos = 2-dicyclohexylphosphin-2',4',6'-triisopropylbiphenyl, Ac = acetyl, THF = tetrahydrofuran, DMF = *N,N*-dimethylformamide.

Reference Compounds

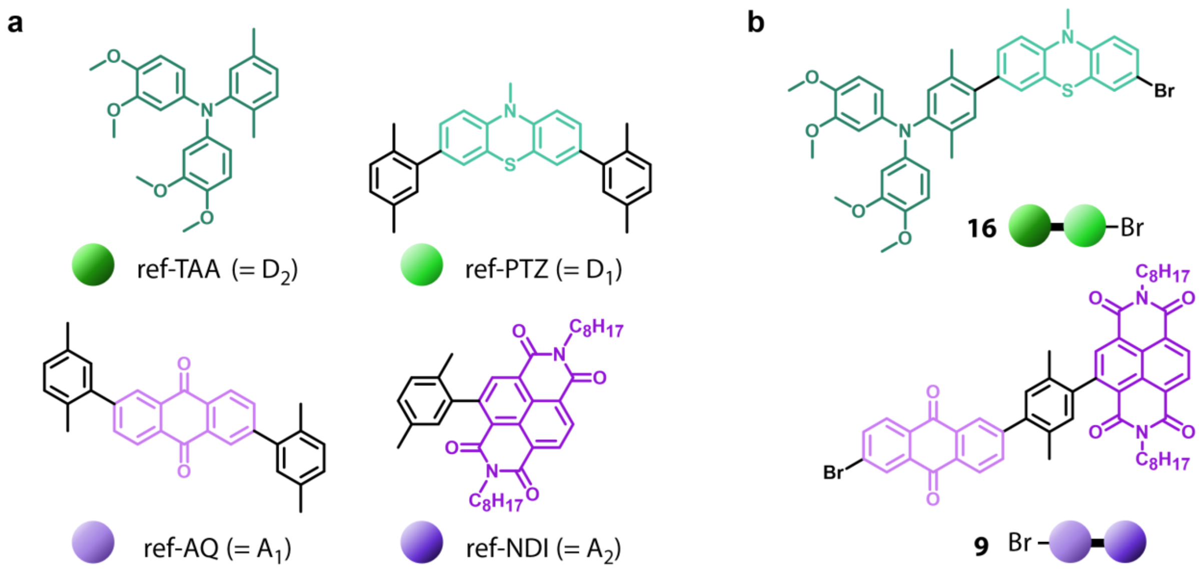

Figure S1: a) Structures of the compounds used as reference molecules for the individual redox units. In the main article, they are used for the cyclic voltammetry in Fig. 2b (top) and for the spectroelectrochemistry in Fig. 3d,e (bottom). b) Structures of the compounds from the NMR experiments in Fig. 2d.

UV-Vis Absorption Spectroscopy

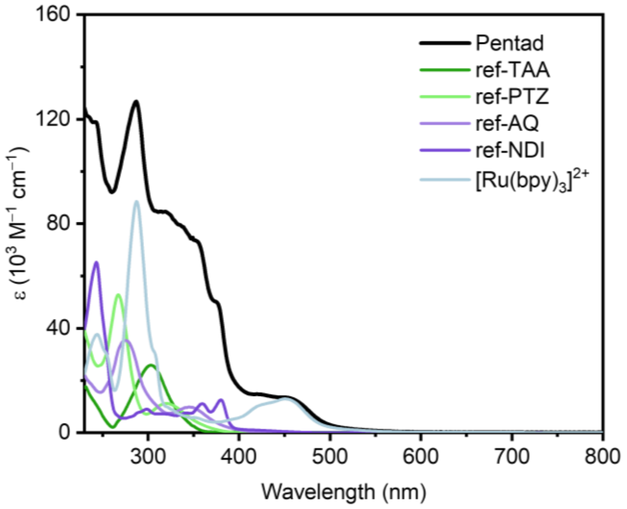

Figure S2: UV-vis absorption spectra of the pentad and the reference compounds from Figure S1a. The spectra of the complexes were recorded in dry MeCN, those of the organic reference molecules in dry CH_2Cl_2.

Cyclic Voltammetry

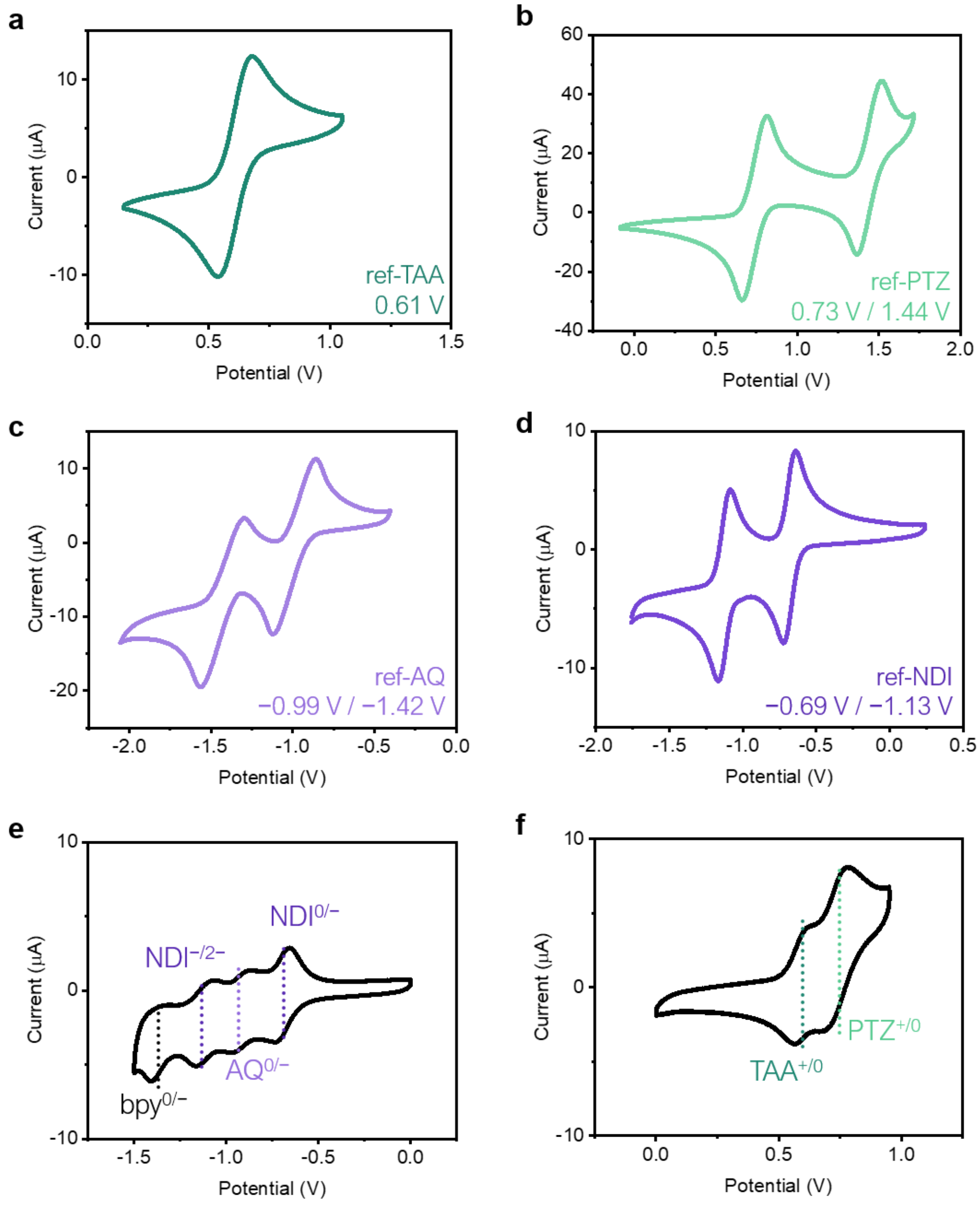

Figure S3: Cyclic voltammograms of a) 1.0 mM ref-TAA, b) 1.0 mM ref-PTZ, c) 1.0 mM ref-AQ, and d) 1.0 mM ref-NDI in dry, Ar saturated CH_2Cl_2 solutions at room temperature. Cyclic voltammograms of e) 0.5 mM pentad in dry, Ar saturated CH_2Cl_2, and f) 0.5 mM pentad in dry, Ar saturated MeCN at room temperature. All cyclic voltammograms are referenced against a saturated calomel electrode (SCE). A glassy carbon disk was used as working electrode and a silver wire served as the counter electrode. The scan rates were 100 mV s^{-1} and tetra-*n*-butylammonium hexafluorophosphate (100 mM) was used as the supporting electrolyte.

Energy Level Diagram

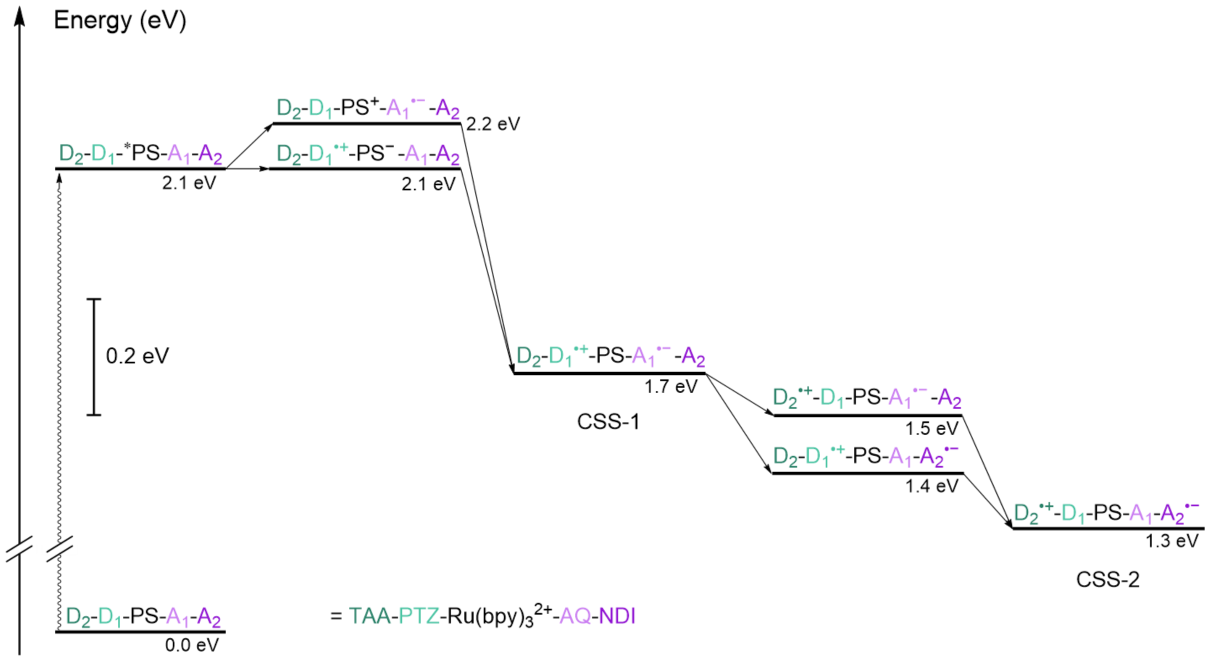

Figure S4: Energy level diagram based on literature redox potentials of $Ru(bpy)_3^{2+}$ [5] and the redox potentials from Table 1 in the main article and Figure S3.

A detailed explanation of the scheme can be found in our recent publication.[1]

Spectroelectrochemistry

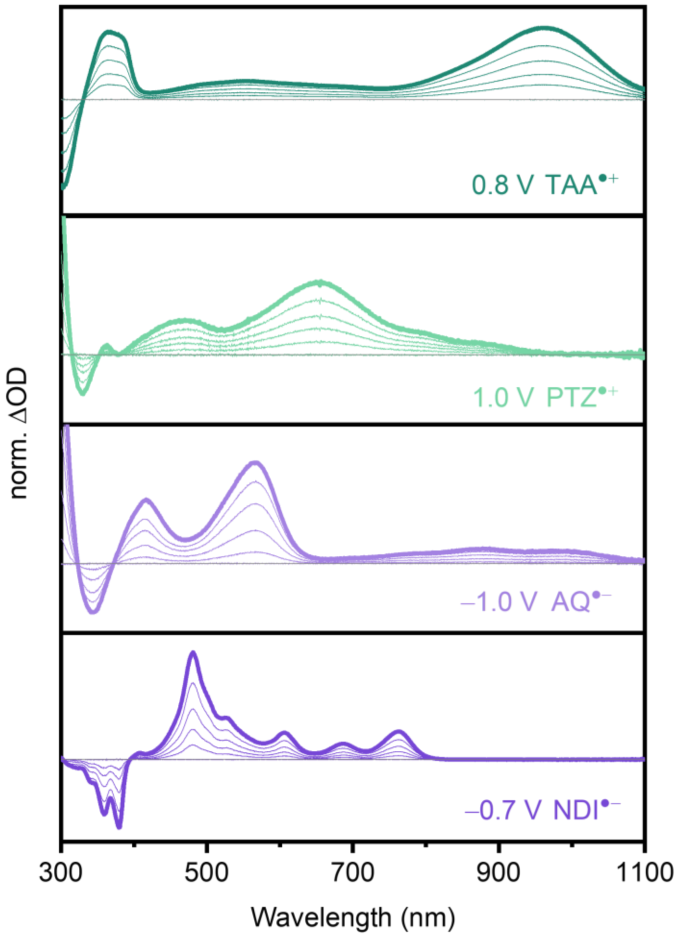

Figure S5: Normalized difference spectra of the oxidized or reduced forms of the reference compounds from Figure S1 obtained during application of the indicated potentials (all vs SCE). 250 µM solutions of the compounds in dry, N_2 saturated CH_2Cl_2 containing 100 mM tetra-*n*-butylammonium hexafluorophosphate were used. In a cuvette with an optical path length of 1.0 mm, a platinum mesh electrode was used as working electrode and a platinum wire as the counter electrode.

Transient Absorption Spectroscopy: Reference Triads

Reference for the Inner Triad (= D_1-PS-A_1):

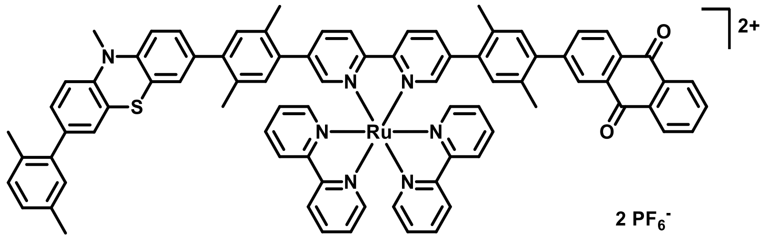

This triad is a reference compound, representing the inner triad of the pentad with only D_1 (PTZ) and A_1 (AQ) present. The synthesis and characterization are described in our recent publication.[1] The spectroscopic features of the charge-separated state observed upon excitation and electron transfers are directly comparable to CSS-1.

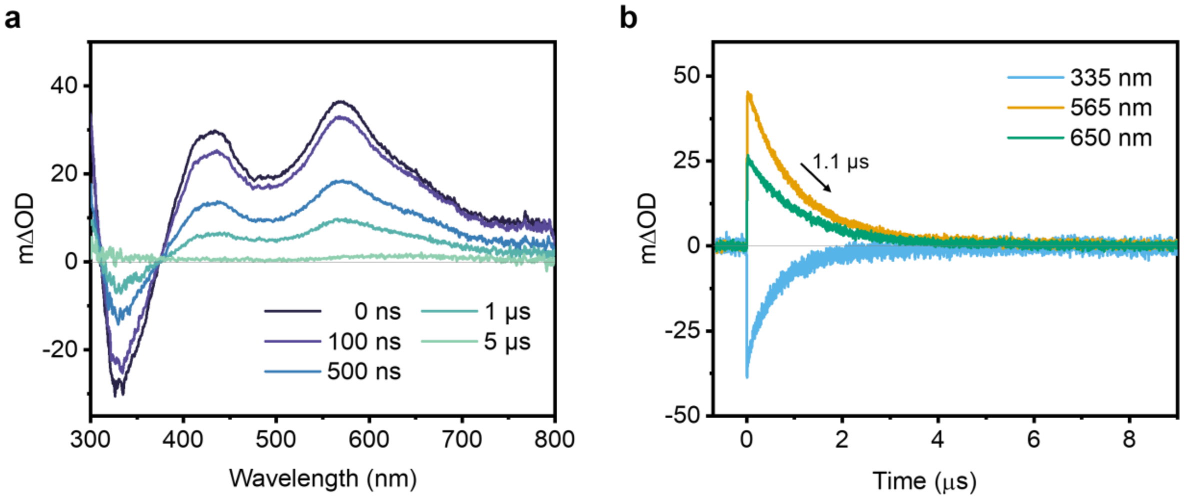

Figure S6: a) Transient absorption spectra of the D_1-PS-A_1 reference triad in dry, Ar saturated MeCN (16 μM) after excitation with 460 nm pulses of ca. 10 ns duration. The spectra are time integrated for 200 ns after the indicated delays. Comparison to the reference spectra in Figure S5 allows to assign the maximum at 560 nm to $AQ^{\bullet -}$ and the shoulder at 650 nm to $PTZ^{\bullet +}$. The maximum at ca. 420 nm has the expected contributions of both $AQ^{\bullet -}$ and $PTZ^{\bullet +}$. b) The decays of the main signals of $AQ^{\bullet -}$ at 565 nm and $PTZ^{\bullet +}$ at 650 nm were monitored together with the recovery of the bleach at 335 nm. A charge-separated state lifetime of 1.1 μs was extracted from single-exponential fitting.

Short Triad:

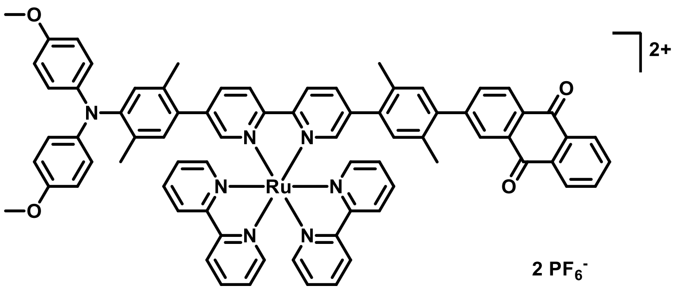

The synthesis and characterization of the short triad using monomethoxy triarylamine (mTAA) as electron donor is already described.[2]

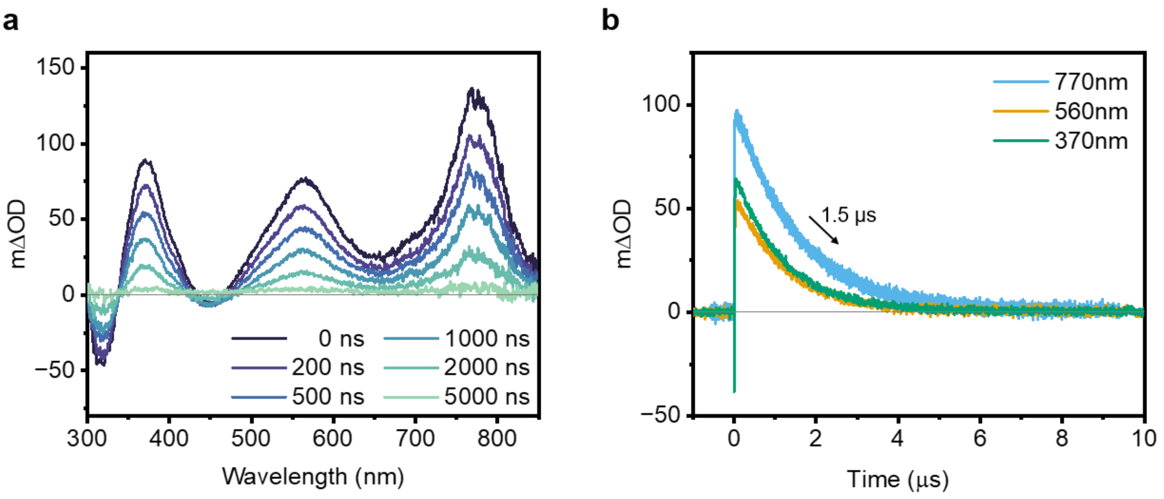

Figure S7: a) Transient absorption spectra of the short triad in dry, Ar saturated MeCN (15 μM) after excitation with 460 nm pulses of ca. 10 ns duration. The spectra are time integrated for 200 ns after the indicated delays. The maxima at 370 nm and 770 nm can be assigned to $mTAA^{\bullet+}$ and the maximum at 560 nm to $AQ^{\bullet-}$.[2] b) The decays of the signals of $AQ^{\bullet-}$ (at 560 nm) and of $mTAA^{\bullet+}$ (at 370 nm and 770 nm) were monitored.

Long Triad:

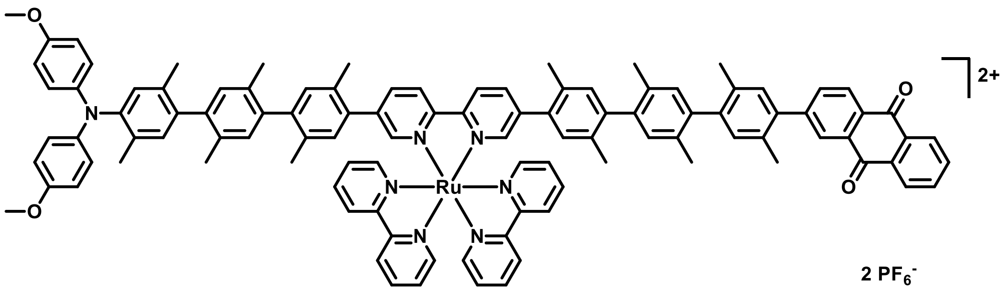

The synthesis and characterization of the long triad is already described.[3]

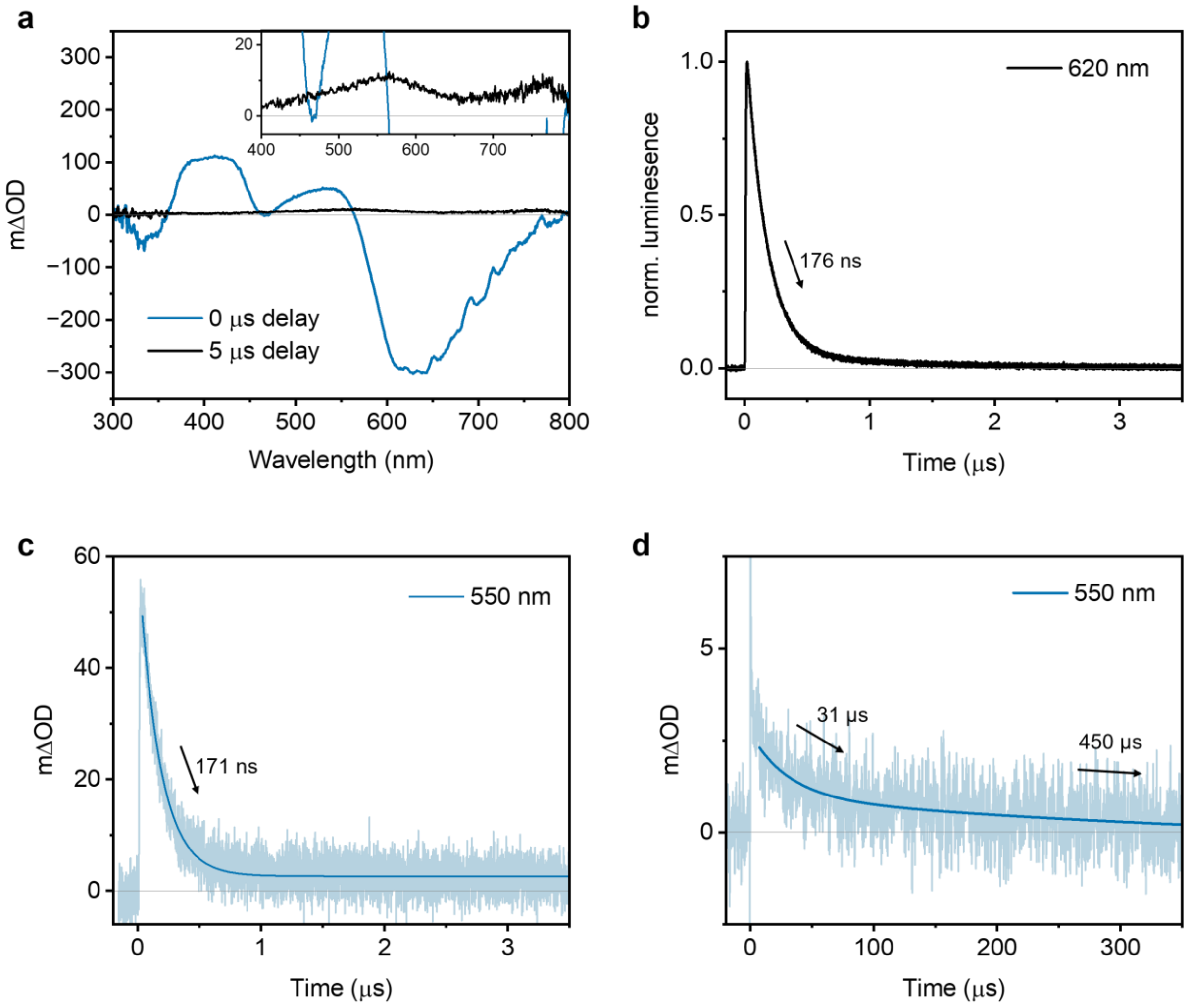

Figure S8: a) Transient absorption spectra of the long triad in dry, Ar saturated MeCN (20 μM) after excitation with 460 nm pulses of ca. 10 ns duration. The two spectra are time-integrated over 200 ns after the indicated delays. b) The decay of the emission observable in (a) at 620 nm is monitored. Exponential fitting provides a lifetime of 176 ns for the emissive excited state. c) The decay of the transient absorption signal

at 550 nm is followed. The initial decay of the transient absorption signal belonging to the emissive excited state decays with 171 ns leaving a longer-lived signal. The 171 ns matches well the decay of the emission and the literature value of 160 ns.[3] d) The long-lived component in (c) decays biexponentially with ca. 30 μs and 450 μs, also matching the literature values of 28 μs and > 400 μs.[3]

As already discussed in detail,[3,6] the long distance between the photosensitizer and the electron donor and acceptor results in a slow quenching of the excited state. Compared to the other compounds presented here, this allows to observe luminescence from the decay of the initially excited state (Figure S8b). The same decay is also observed in the intense excited state absorption signals at 400 nm and 550 nm (blue trace, Figure S8a) that decay with the same rate (Figure S8c). After ca. 1.5 μs, only the charge-separated state is left (black trace, Figure S8a) with the same spectroscopic features as the short triad at 560 nm and 770 nm. This CSS then recombines and has a lifetime of ca. 30 μs in Ar saturated MeCN at 20 °C. In a MeCN/H_2O (1:1 v/v) mixture, hydrogen bonds to $AQ^{\bullet-}$ can stabilize it on longer timescales.[3] The slow decay component with a lifetime of 450 μs is attributed to bimolecular processes (Figure S8d).[3]

Relative Actinometry: Pentad

The procedure is described in detail in the Supplementary Information of our recent publication.[1]

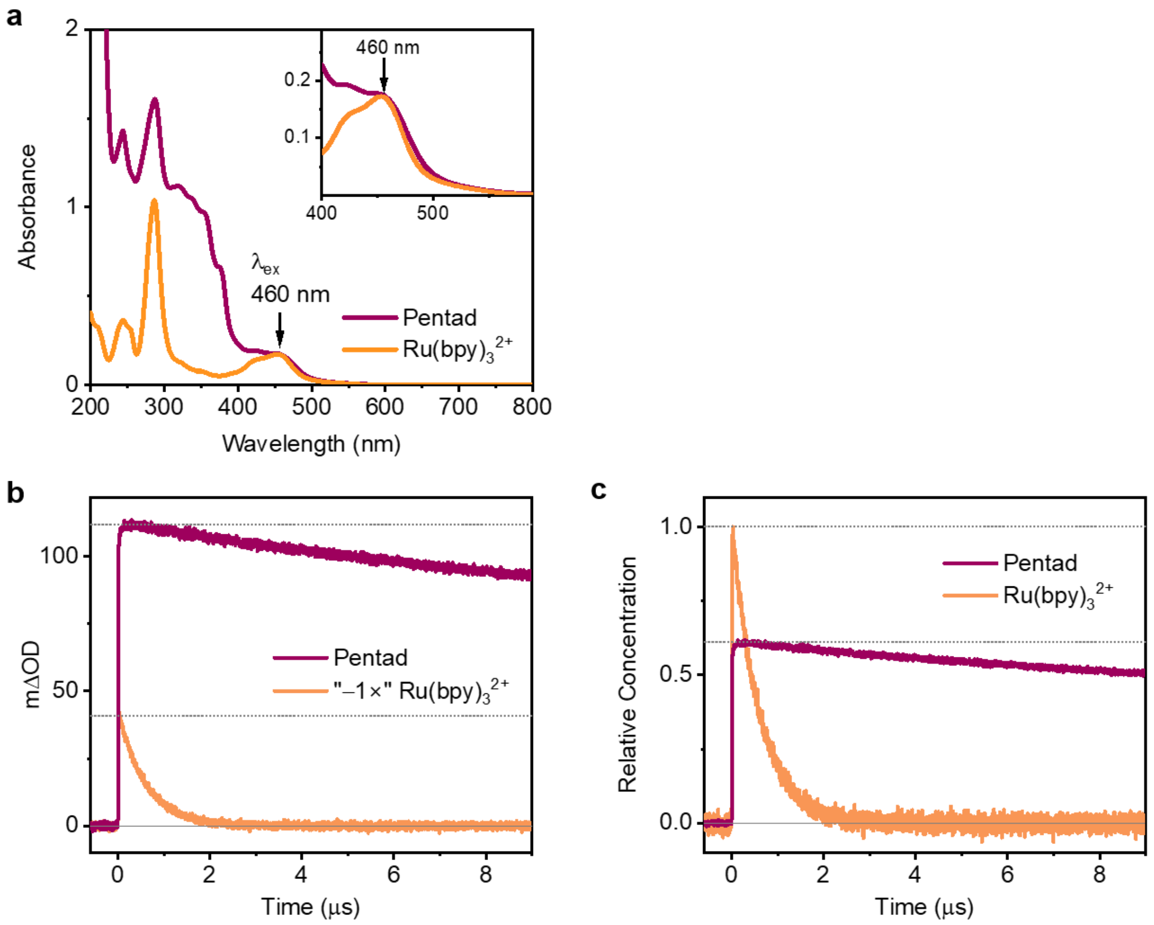

Figure S9: a) UV-vis absorption spectra of the pentad (purple) in dry, Ar saturated MeCN and $Ru(bpy)_3Cl_2$ (orange) in Ar saturated H_2O. The concentrations of the two samples were adjusted to have the same absorbance at the excitation wavelength of 460 nm. Without stopping the laser between the measurements, the two samples were excited with 460 nm pulses of ca. 10 ns duration. b) The decay of the transient absorption signal of the pentad at 476 nm (purple) and the recovery of the ground state bleach of the $Ru(bpy)_3^{2+}$ reference at 455 nm (orange) were recorded. c) Dividing the data from (b) by the respective changes in molar extinction coefficient, $\Delta\varepsilon_{476\ nm}$ = 46'600 $M^{-1}\ cm^{-1}$ for $NDI^{\bullet-}$ and $\Delta\varepsilon_{455\ nm}$ = −10'450 $M^{-1}\ cm^{-1}$ for ^{3}MLCT-excited $Ru(bpy)_3^{2+}$,[1,7] and normalization to the maximum of $Ru(bpy)_3^{2+}$ provides relative concentration profiles. This provides a charge separation quantum yield of 61% for the pentad.

Relative Actinometry: Short Triad and Long Triad

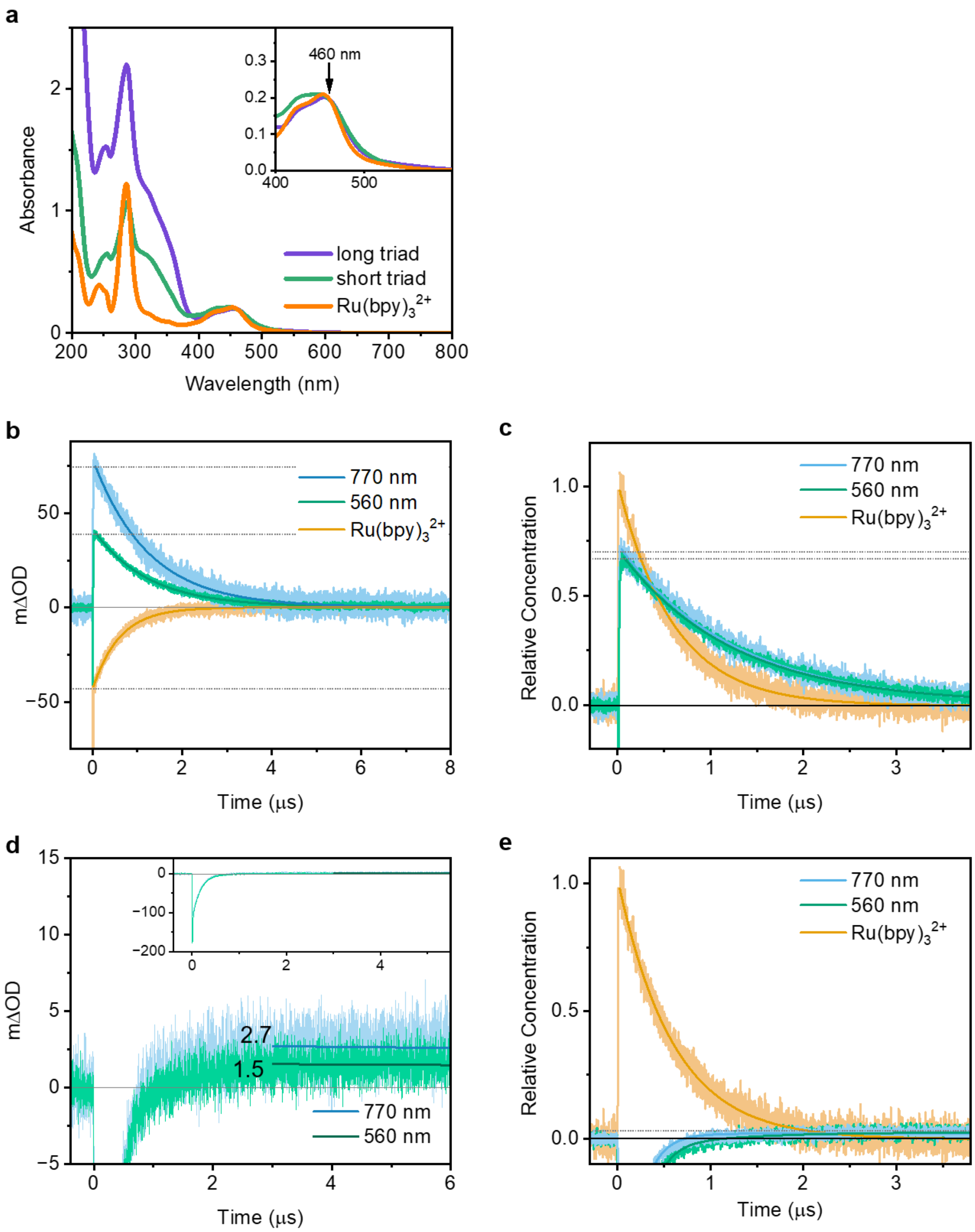

Figure S10: a) UV-vis absorption spectra of the short triad (green) and the long triad (violet) in dry, Ar saturated MeCN and $Ru(bpy)_3Cl_2$ (orange) in Ar saturated H_2O. The concentrations of the three samples were adjusted to have the same absorbance at the excitation wavelength of 460 nm. Without stopping the laser between the

measurements, the three samples were excited with 460 nm pulses of ca. 10 ns duration. b) The decays of the transient absorption signal of the short triad at 560 nm (green) and 770 nm (blue), and the recovery of the ground state bleach of the $Ru(bpy)_3^{2+}$ reference at 450 nm (orange) were recorded. c) Dividing the data from (b) by the respective changes in molar extinction coefficient, $\Delta\varepsilon_{560\ nm}$ = 15’100 M^{-1} cm^{-1} for $AQ^{\bullet-}$,[8] $\Delta\varepsilon_{770\ nm}$ = 27’500 M^{-1} cm^{-1} for $mTAA^{\bullet+}$,[8] and $\Delta\varepsilon_{450\ nm}$ = −11’000 M^{-1} cm^{-1} for ^{3}MLCT-excited $Ru(bpy)_3^{2+}$,[7] and normalization to the maximum of $Ru(bpy)_3^{2+}$ provides relative concentration profiles. This procedure results in a charge separation quantum yield of 67% for the $AQ^{\bullet-}$ signal and 69% for the $mTAA^{\bullet+}$ signal. On average, this gives a charge separation quantum yield of 68% for the short triad. d) The decays of the transient absorption signal of the long triad at 560 nm (green) and 770 nm (blue) were recorded. The relevant changes in optical density were determined by linear fitting the data after 3 μs, due to the low signal to noise ratio. The changes in optical density were 1.5×10^{-3} for the 560 nm trace and 2.7×10^{-3} for the 770 nm trace. Importantly, the ratio between the two signals of 1.8 is very similar to the short triad (ratio = 1.9). e) Dividing the data from (d) by the respective changes in molar extinction coefficient, $\Delta\varepsilon_{560\ nm}$ = 15’100 M^{-1} cm^{-1} for $AQ^{\bullet-}$, $\Delta\varepsilon_{770\ nm}$ = 27’500 M^{-1} cm^{-1} for $mTAA^{\bullet+}$, and $\Delta\varepsilon_{450\ nm}$ = −11’000 M^{-1} cm^{-1} for ^{3}MLCT-excited $Ru(bpy)_3^{2+}$,[7] and normalization to the maximum of $Ru(bpy)_3^{2+}$ provides relative concentration profiles. This procedure results in a charge separation quantum yield of 3% for the $AQ^{\bullet-}$ signal and 3% for the $mTAA^{\bullet+}$ signal. On average, this gives a charge separation quantum yield of 3% for the long triad.

	**short triad**	**long triad**	$Ru(bpy)_3^{2+}$
$\Delta\varepsilon_{560\ nm}$ ($AQ^{\bullet-}$)	15’100 M^{-1} cm^{-1}		$\Delta\varepsilon_{450\ nm}$ ($^{*}Ru(bpy)_3^{2+}$): −11’000 M^{-1} cm^{-1}
$\Delta\varepsilon_{770\ nm}$ ($mTAA^{\bullet+}$)	27’500 M^{-1} cm^{-1}		
initial mΔOD at 560 nm	39.3	1.5	mΔOD at 450 nm: −42.8
initial mΔOD at 770 nm	74.3	2.7	
ratio (770 nm / 560 nm)	1.9	1.8	
Φ_{CSS} (from the 560 nm trace)	67%	2.6%	
Φ_{CSS} (from the 770 nm trace)	69%	2.5%	
Average	68%	3%	

Correction for Luminescence

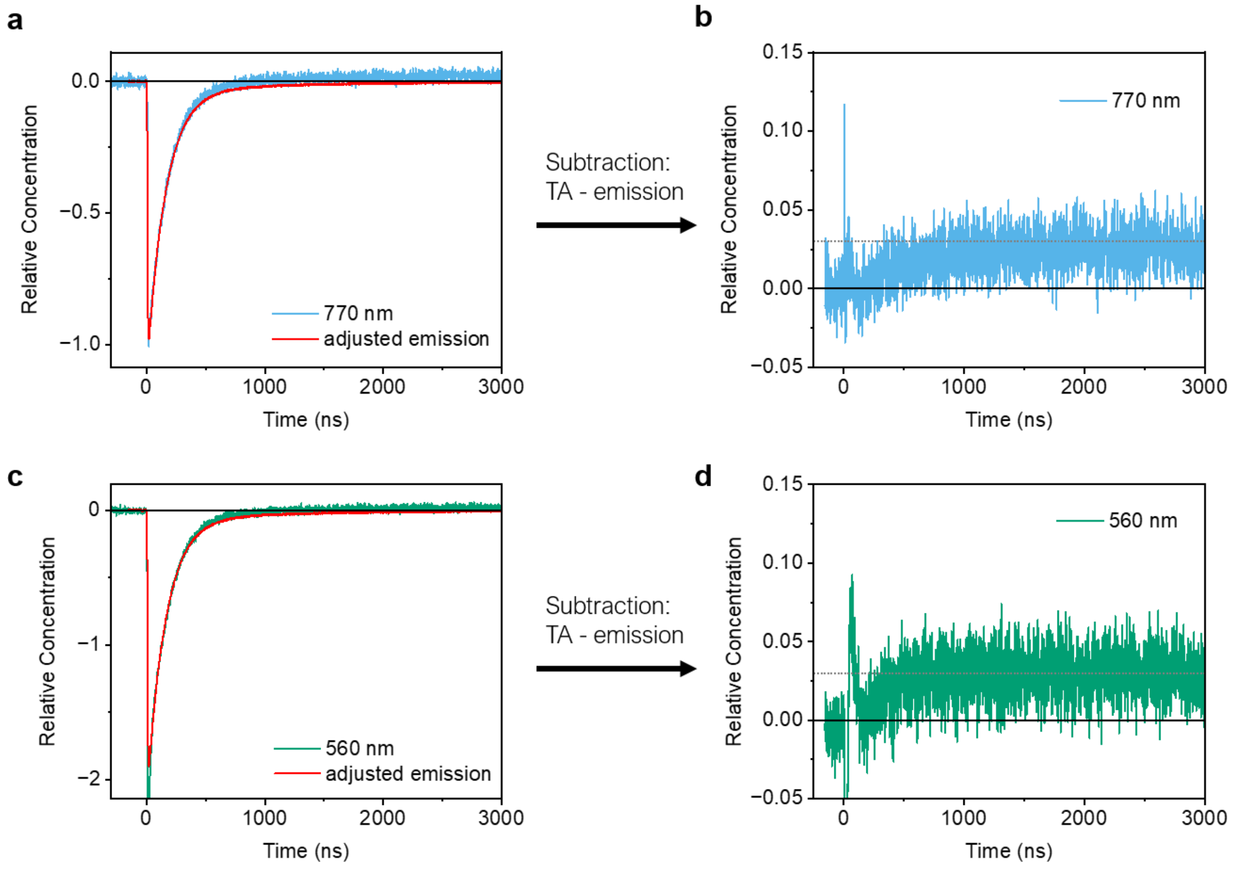

Figure S11: a,c) The relative concentration traces of the long triad from Figure S10e together with the luminescence decay (red, from Figure S8b), which is adjusted in intensity to match the early decay behavior. b,d) Subtraction of the emission decay trace from the transient absorption signal provides the concentration profiles of the long triad that are corrected for the emission. Hence, it now only represents the contribution of the charge-separated state. The trace in (d) is used for Figure 4f of the main article.

DFT

Ground-state geometry of the main ligand of the pentad was optimized using Density Functional Theory (DFT) at the B3LYP/6-31G* level of theory. Molecular orbitals were calculated based on the optimized geometry and used for visualization purposes.

Atom	x	y	z	Atom	x	y	z	Atom	x	y	z
O	-10.092347	-10.488847	-1.833503	C	12.153157	13.66545	-0.978723	H	12.875354	12.925855	0.89504
C	-9.134723	-10.320655	-1.085265	N	13.463545	13.743586	-1.538324	H	8.888445	14.202882	-1.684717
C	-8.650498	-11.411685	-0.187579	C	13.967854	14.998218	-1.97333	H	11.485758	15.546578	-3.226595
C	-7.55313	-11.214239	0.672683	C	13.568853	16.180512	-1.325099	H	11.822605	13.897196	-3.730472
C	-7.138535	-12.261728	1.50238	C	14.019847	17.425508	-1.769072	H	10.142974	14.471005	-3.649232
C	-7.801143	-13.482454	1.475062	O	13.652154	18.617907	-1.211356	H	12.898767	16.108303	-0.477805
C	-8.899472	-13.699052	0.61834	C	12.694813	18.60249	-0.164759	H	11.754685	18.136341	-0.487319
C	-9.605895	-15.009709	0.641831	C	14.920231	17.499473	-2.85356	H	13.070646	18.075395	0.722396
C	-9.97807	-15.531142	1.892072	O	15.345103	18.707323	-3.357082	H	12.512869	19.648694	0.088264
C	-10.616703	-16.759401	2.048411	C	16.256516	19.43215	-2.530514	H	16.507297	20.342255	-3.081067
C	-10.964885	-17.270405	3.425223	C	15.321718	16.320346	-3.476235	H	15.803013	19.698973	-1.570504
C	-9.893306	-15.754611	-0.520773	C	14.84662	15.077569	-3.060823	H	17.172939	18.85194	-2.354141
C	-9.512787	-15.303902	-1.914234	C	14.349771	12.640952	-1.363911	H	15.997039	16.399841	-4.323106
C	-10.537887	-16.987141	-0.362853	C	13.882739	11.339399	-1.635934	H	15.152654	14.17261	-3.574479
C	-10.888537	-17.504664	0.88607	C	14.707828	10.233422	-1.466138	H	12.862428	11.215202	-1.97699
C	-11.63072	-18.805474	0.934632	O	14.334203	8.943834	-1.700521	H	12.791967	9.189404	-3.087586
C	-11.055268	-20.054054	1.20267	C	13.003437	8.704029	-2.125137	H	12.91804	7.621865	-2.240236
C	-11.861426	-21.227927	1.18553	C	16.048585	10.412145	-1.037356	H	12.272673	9.049945	-1.381754
C	-11.307128	-22.511486	1.424901	O	16.786805	9.271087	-0.908719	H	18.181909	9.848389	0.533854
C	-9.854984	-22.671718	1.688486	C	18.125599	9.398956	-0.467346	H	18.526247	8.384032	-0.429438
N	-9.097915	-21.494952	1.68956	C	16.505245	11.701639	-0.782569	H	18.727491	10.00006	-1.163109
C	-7.65646	-21.594015	1.95358	C	15.66258	12.811052	-0.929979	H	17.524239	11.862002	-0.449247
C	-9.599199	-20.196036	1.500426	C	7.682005	12.164532	1.237325	H	16.038668	13.803717	-0.708496
O	-8.833322	-19.248447	1.585525	C	6.374841	12.086405	1.710033	H	8.296766	11.26964	1.255021
O	-9.344747	-23.764488	1.888962	S	5.787465	10.574437	2.450886	H	3.803908	7.21768	-0.31204
C	-12.110465	-23.641146	1.408872	C	1.657773	7.347063	0.038253	H	2.808005	6.932595	-1.738505
C	-13.488471	-23.539128	1.153173	C	2.886519	7.532104	-0.825692	H	3.026791	8.578352	-1.114121
C	-14.056884	-22.300358	0.909073	C	0.85717	6.218722	-0.152738	H	1.15817	5.492959	-0.905314
C	-13.254646	-21.134615	0.917875	C	-8.160331	-6.746318	-1.826675	H	-8.48592	-5.9384	-2.476087
C	-13.821027	-19.86725	0.652952	C	-8.828002	-7.96598	-1.8537	H	-9.677774	-8.127215	-2.509119
C	-15.275073	-19.734646	0.362304	C	-8.408425	-9.020915	-1.035367	N	-3.945671	0.636352	0.589877

Atom	x	y	z	Atom	x	y	z	Atom	x	y	z
N	-16.030098	-20.91737	0.39639	O	-5.860741	-9.751977	1.460572	C	-4.590727	-0.517391	0.430358
C	-17.470456	-20.825981	0.125325	H	-6.288017	-12.097517	2.155991	C	-4.481723	-1.348814	-0.698948
C	-15.513626	-22.200439	0.628544	H	-7.460434	-14.294884	2.110561	C	-3.618664	-0.90738	-1.710249
O	-16.244699	-23.179077	0.601837	H	-9.769572	-14.939296	2.78005	H	-5.225689	-0.819515	1.261622
O	-15.792887	-18.656673	0.113257	H	-10.824197	-16.489986	4.179202	H	-3.48447	-1.506044	-2.607653
C	-13.020187	-18.742979	0.654398	H	-10.321959	-18.116584	3.697097	C	-0.309442	5.96499	0.583677
C	-9.30965	-12.643752	-0.20708	H	-9.423429	-16.165831	-2.583162	C	1.259616	8.281279	1.020174
C	-6.822521	-9.918404	0.717221	H	-8.560046	-14.766146	-1.92448	C	-2.939066	0.296883	-1.561567
C	-7.304264	-8.829393	-0.182499	H	-10.753092	-17.577267	-1.250982	H	-2.252248	0.634049	-2.331687
C	-6.640528	-7.601208	-0.163938	H	-7.401954	-22.650137	2.000066	C	-2.413405	2.342779	-0.185632
C	-7.049297	-6.542472	-0.985084	H	-7.414908	-21.101582	2.899021	C	-3.128383	1.051229	-0.393833
C	-6.359676	-5.223178	-0.942896	H	-11.646623	-24.603628	1.595618	C	-2.116597	3.192414	-1.261742
C	-4.967448	-5.086903	-1.132526	H	-14.123015	-24.418683	1.137938	H	-2.434261	2.940609	-2.268886
C	-4.055394	-6.255659	-1.437113	H	-17.767899	-19.786085	0.241056	C	-1.4419	4.384269	-1.019698
C	-4.423833	-3.802722	-1.055575	H	-18.003532	-21.471308	0.823466	H	-1.219944	5.065168	-1.837256
C	-7.128014	-4.073043	-0.712839	H	-13.475245	-17.782223	0.438103	C	-1.071151	4.722464	0.288658
C	-6.581236	-2.790092	-0.622646	H	-10.162316	-12.749802	-0.868344	C	-1.419336	3.796987	1.289149
C	-7.499835	-1.612086	-0.38074	H	-5.808914	-7.486353	0.522665	H	-1.133922	3.988996	2.32211
C	-5.185809	-2.655914	-0.787759	H	-3.144653	-5.908662	-1.935362	N	-2.065106	2.652258	1.075513
C	3.390459	9.502719	1.61784	H	-3.744153	-6.78418	-0.527493	C	2.023105	9.5292	1.299828
C	4.089679	10.669409	1.912076	H	-4.536874	-6.993128	-2.086676	C	0.079534	8.039376	1.738197
C	3.45519	11.926259	1.860032	H	-3.351165	-3.686607	-1.192961	H	-0.225855	8.770182	2.483771
N	4.192306	13.098412	2.139676	H	-8.201581	-4.188016	-0.580747	C	-0.717318	6.906182	1.553345
C	3.459355	14.323923	2.41338	H	-7.16601	-0.717795	-0.915636	C	-1.983675	6.755245	2.368401
C	5.518834	13.203756	1.660009	H	-8.51769	-1.845262	-0.709694	H	-1.834581	6.122154	3.252194
C	6.029164	14.394836	1.12502	H	-7.558156	-1.346983	0.682453	H	-2.792841	6.303749	1.786059
C	7.35002	14.473004	0.684127	H	3.914786	8.55354	1.672508	H	-2.326236	7.731118	2.727795
C	8.205962	13.3647	0.72966	H	4.122374	15.026457	2.923817	C	1.391487	10.779225	1.271197
C	9.59286	13.45159	0.194591	H	3.06058	14.812865	1.510269	H	0.338796	10.838802	1.009258
C	10.733815	13.071019	0.936294	H	2.624906	14.095934	3.081045	C	2.093621	11.956943	1.525953
C	10.658604	12.58355	2.367158	H	5.396254	15.271874	1.046467	H	1.572905	12.904574	1.445261
C	11.986369	13.195504	0.331027	H	7.722458	15.415505	0.29268	H	-12.00382	-17.6142	3.488181
C	9.77286	13.936302	-1.110426	H	11.601493	12.777625	2.888834	H	-10.264855	-14.634127	-2.350344
C	11.019547	14.042668	-1.730087	H	9.853093	13.073645	2.92226	H	-7.104396	-21.094573	1.155109
C	11.120776	14.514187	-3.159055	H	10.477544	11.502352	2.426862	H	-17.686653	-21.15962	-0.89418

References

[1] M. Brändlin, B. Pfund, O. S. Wenger, *Nat. Chem.* **2025**, doi.org/10.1038/s41557-025-01912-x.

[2] J. Hankache, M. Niemi, H. Lemmetyinen, O. S. Wenger, *Inorg. Chem.* **2012**, *51*, 6333–6344.

[3] M. Kuss-Petermann, O. S. Wenger, *Angew. Chem. Int. Ed.* **2016**, *55*, 815–819.

[4] G. R. Fulmer, A. J. M. Miller, N. H. Sherden, H. E. Gottlieb, A. Nudelman, B. M. Stoltz, J. E. Bercaw, K. I. Goldberg, *Organometallics* **2010**, *29*, 2176–2179.

[5] C. K. Prier, D. A. Rankic, D. W. C. MacMillan, *Chem. Rev.* **2013**, *113*, 5322–5363.

[6] M. Kuss-Petermann, O. S. Wenger, *J. Am. Chem. Soc.* **2016**, *138*, 1349–1358.

[7] P. Müller, K. Brettel, *Photochem. Photobiol. Sci.* **2012**, *11*, 632–636.

[8] S. Neumann, C. Kerzig, O. S. Wenger, *Chem. Sci.* **2019**, *10*, 5624–5633.
